# The Lipopolysaccharide Core of *Brucella abortus* Acts as a Shield Against Innate Immunity Recognition

**DOI:** 10.1371/journal.ppat.1002675

**Published:** 2012-05-10

**Authors:** Raquel Conde-Álvarez, Vilma Arce-Gorvel, Maite Iriarte, Mateja Manček-Keber, Elías Barquero-Calvo, Leyre Palacios-Chaves, Carlos Chacón-Díaz, Esteban Chaves-Olarte, Anna Martirosyan, Kristine von Bargen, María-Jesús Grilló, Roman Jerala, Klaus Brandenburg, Enrique Llobet, José A. Bengoechea, Edgardo Moreno, Ignacio Moriyón, Jean-Pierre Gorvel

**Affiliations:** 1 Institute for Tropical Health and Departamento de Microbiología y Parasitología, Universidad de Navarra, Pamplona, Spain; 2 Focal Area Infection Biology, Biozentrum of the University of Basel, Basel, Switzerland; 3 Centre d'Immunologie de Marseille-Luminy (CIML), Aix-Marseille University UM2 Marseille France, Institut National de la Santé et de la Recherche Médicale (INSERM) U1104 Marseille France, Centre National de la Recherche Scientifique (CNRS) UMR7280 Marseille France; 4 Department of Biotechnology, National Institute of Chemistry, Hajdrihova, Ljubljana, Slovenia; 5 Programa de Investigación en Enfermedades Tropicales, Escuela de Medicina Veterinaria, Universidad Nacional, Heredia, Costa Rica; 6 Centro de Investigación en Enfermedades Tropicales, Universidad de Costa Rica, San José, Costa Rica; 7 Instituto de Agrobiotecnología CSIC-UPNA-Gobierno de Navarra, Pamplona, Spain; 8 Forschungzentrum Borstel, Borstel, Germany; 9 Laboratory Microbial Patogénesis, Fundación Investigación Sanitaria Illes Balears-CSIC, Bunyola, Spain; 10 Instituto Clodomiro Picado, Universidad de Costa Rica, San José, Costa Rica; University of California, Davis, United States of America

## Abstract

Innate immunity recognizes bacterial molecules bearing pathogen-associated molecular patterns to launch inflammatory responses leading to the activation of adaptive immunity. However, the lipopolysaccharide (LPS) of the gram-negative bacterium *Brucella* lacks a marked pathogen-associated molecular pattern, and it has been postulated that this delays the development of immunity, creating a gap that is critical for the bacterium to reach the intracellular replicative niche. We found that a *B. abortus* mutant in the *wadC* gene displayed a disrupted LPS core while keeping both the LPS O-polysaccharide and lipid A. In mice, the *wadC* mutant induced proinflammatory responses and was attenuated. In addition, it was sensitive to killing by non-immune serum and bactericidal peptides and did not multiply in dendritic cells being targeted to lysosomal compartments. In contrast to wild type *B. abortus*, the *wadC* mutant induced dendritic cell maturation and secretion of pro-inflammatory cytokines. All these properties were reproduced by the *wadC* mutant purified LPS in a TLR4-dependent manner. Moreover, the core-mutated LPS displayed an increased binding to MD-2, the TLR4 co-receptor leading to subsequent increase in intracellular signaling. Here we show that *Brucella* escapes recognition in early stages of infection by expressing a shield against recognition by innate immunity in its LPS core and identify a novel virulence mechanism in intracellular pathogenic gram-negative bacteria. These results also encourage for an improvement in the generation of novel bacterial vaccines.

## Author Summary

Brucellosis is one of the most extended bacterial zoonosis in the world and an important cause of economic losses and human suffering. The causative agents belong to the genus *Brucella*, a group of highly infectious gram-negative bacteria characterized by their ability to escape early detection by innate immunity. This stealthy behavior effectively delays the development of immunity, creating a gap that is used by the bacterium to penetrate into a variety of cells and to activate complementary virulence mechanisms such as the type IV secretion system. By this manner, the brucellae divert intracellular trafficking to reach a safe multiplication niche and establish chronic infections. Our results show that an inner section of the *Brucella* LPS (a molecule that in most bacteria is detected by innate immunity), effectively contributes to block recognition by soluble molecules and cellular receptors of the host innate immune system. Accordingly, a mutation disrupting the inner but no other lipopolysaccharide sections generates attenuation by impairing the stealthiness characteristics of this pathogen. This is the first *Brucella* mutant in which attenuation is specifically linked to the bolstering of immunity against this pathogen. Therefore, this new virulence mechanism opens the way for the development of improved bacterial vaccines.

## Introduction

Innate immunity plays a fundamental role in the defense against microorganisms. In addition to the passive action of physical and physicochemical barriers, the effectiveness of innate immunity relies on pathogen recognition receptors that quickly perceive the presence of invaders. Upon binding to microbial molecules bearing pathogen-associated molecular patterns (PAMP), pathogen recognition receptors trigger a cascade of signals that include the release of proinflammatory mediators, which in turn may activate adaptive immunity. Cells like macrophages and dendritic cells are equipped with a variety of pathogen recognition receptors, which can be activated by bacterial PAMP such as lipoproteins, glycolipids, peptidoglycan or DNA. However, some bacteria are able to generate chronic infections by residing and multiplying in these host cells. A relevant model of this kind of pathogens is represented by the genus *Brucella* [1], a group of α-*Proteobacteria* that have a great impact on animal and human health worldwide, and whose virulence relies in part upon the failure of pathogen recognition receptors to sense *Brucella* during the initial stages of infection [2], [3].

*Brucella* surface lipoproteins, ornithine lipids, flagellum-like structures and the LPS do not bear a marked PAMP [2], [3], [4], [5]. The most conspicuous PAMP bearing component of the surface of gram-negative bacteria is LPS, also known as endotoxin, a molecule made of three sections: lipid A, core oligosaccharide and O-polysaccharide (O-PS). Typically, LPS express a lipid A made of a glucosamine disaccharide linked predominantly to C12 to C14 acyl chains in ester, amide and acyl-oxyacyl bonds. This structure carries a characteristic PAMP that is recognized by the TLR4-MD2 receptor-coreceptor complex, triggering potent proinflammatory responses that may lead to endotoxic shock. Since *Brucella* lipid A (a diaminoglucose disaccharide substituted with C16, C18, C28 and other very long acyl chains [6]) structurally departs from the canonical lipid A recognized by TLR4-MD2 [7], it is postulated to play a key role in the stealthy behavior of this pathogen; indeed *Brucella* LPS is poorly endotoxic [2], [4], [8], [9]. In addition, the O-PS characteristic of smooth brucellae like *B. abortus*, *B. melitensis* or *B. suis* confers serum and complement resistance, a property not uncommon in the O-PS of gram-negative pathogens, and also modulates the entry into cells [1]. It is not known whether the LPS core sugar structure of *Brucella* or any other gram-negative intracellular pathogen has a direct role in intracellular virulence. Indeed, mutants of smooth *Brucella* affected in the LPS core show different degrees of attenuation, but these results cannot be unambiguously interpreted because all core mutants described so far simultaneously lack the O-chain. (i.e., are rough [R] mutants) and thus are attenuated [10]. Here, we report that mutation of a hitherto unidentified *Brucella* LPS core glycosyltransferase gene generates attenuation without affecting the assembly and linkage of the O-PS or the lipid A section. This attenuation is not caused by a physiological defect associated with a damage of the envelope properties but rather by the removal of a core section that hampers recognition by complement, bactericidal peptides and TLR4-MD2, thus representing a novel virulence mechanism.

## Results

### The *B. abortus wadC* glycosyltransferase gene is required for the synthesis of a core section of smooth LPS

**Figure 1:**
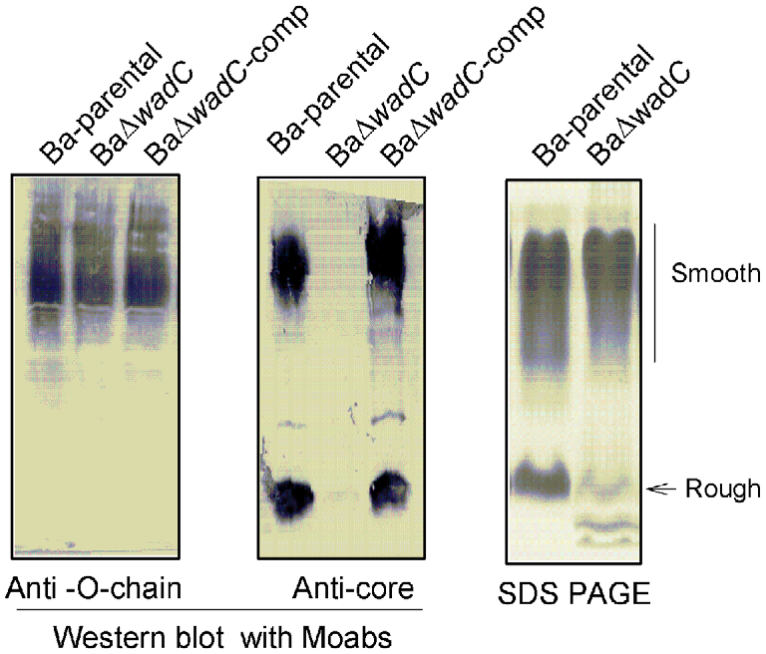
Ba*ΔwadC* carries a partially defective LPS core oligosaccharide. Western blot analyses were performed with monoclonal antibodies Cby-33H8 (O-polysaccharide C/Y epitope) and A68/24D08/G09 (core oligosaccharide) and SDS-proteinase K LPS extracts of Ba-parental, BaΔ*wadC* and BaΔ*wadC*-compl (complemented mutant), and the SDS-PAGE electropherogram of LPS phenol-water extracts was silver stained.

Up to now, only one *Brucella* core glycosyltransferase has been identified [20]. Since LPS core structures are often conserved in phylogenetically related organisms, we scanned the genomes of α-*Proteobacteria* looking for orthologues of glycosyltransferases not involved in O-PS synthesis. We identified *B. abortus* ORF BAB1_1522 as encoding an orthologue (78% similarity) of the *Rhizobium leguminosarum* core mannosyltransferase LpcC [11] and named it *wadC* following accepted nomenclature [12]. We then constructed a non-polar mutant (BaΔ*wadC*; Figure S1) of virulent *B. abortus* 2308 Nal^R^ (Ba-parental) [9]. This mutant showed the same dye and phage sensitivity pattern as the parental strain, and its growth rate in bacteriological media was similarly unaffected (Figure S2). In addition, the mutant reacted normally with polyclonal antibodies to the O-PS and was smooth by the crystal violet-exclusion and acriflavine tests, suggesting the presence of a typical smooth LPS (S-LPS). Thus, for a better analysis of possible LPS defects, we extracted the Ba-parental and BaΔ*wadC* LPSs using the phenol-water protocol [13], [14]. SDS-PAGE and Western-blots with anti-O-PS and anti-core monoclonal antibodies showed that the wild type LPS of the parental Ba-parental strain consisted of both S and R fractions, as expected (Figure 1). However, the BaΔ*wadC* LPS extracts showed a different migration pattern suggesting a core defect. This peculiarity was confirmed by its lack of reactivity of the anti-core monoclonal antibodies A68/24D08/G09 (Figure 1), A68/24G12/A08 and A68/3F03/D5 (Figure S3). The implication of *wadC* was confirmed by complementation with plasmid p*wadC* (strain BaΔ*wadC*-comp) (Figure 1). In addition, the lipids A of both Ba-parental and Ba*ΔwadC* were dominated by molecules carrying the very long chain fatty acids typical of *Brucella* LPS, with minor and not consistent differences in the intensity of some peaks as detected by mass spectrometry (Figure S4).

### BaΔ*wadC* mutant is attenuated and induces proinflammatory responses

**Figure 2:**
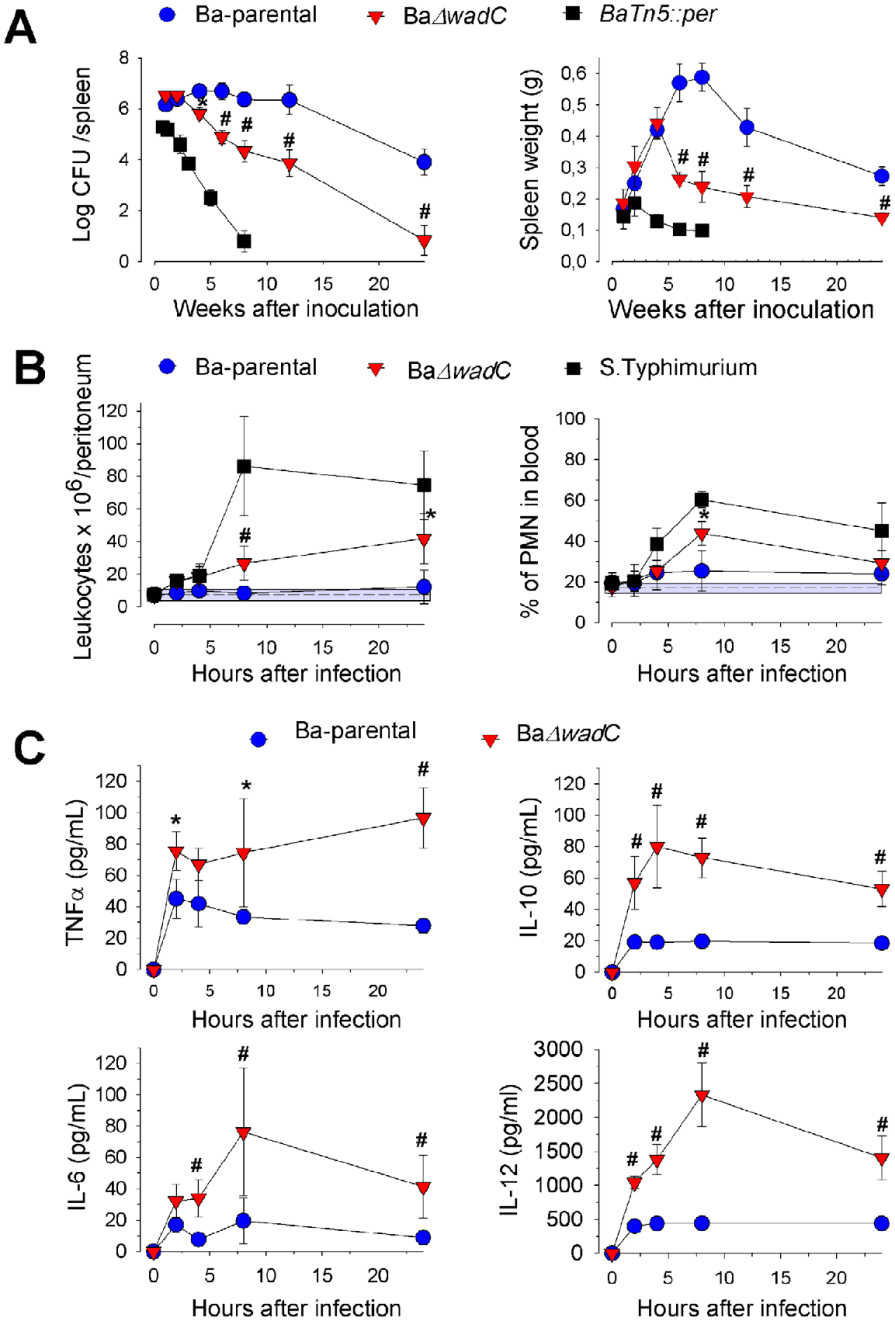
BaΔ*wadC* is attenuated in mice and induces transitory splenomegaly, local leukocyte recruitment and cytokine secretion. (**A**), Left panel, infection kinetics in the spleens of mice intraperitoneally inoculated with 5×10^4^ Ba-parental, BaΔ*wadC* or 1×10^8^ BaTn5::*per*; right panel, spleen weights. (**B**), Leukocyte and PMN levels in the peritoneum (left panel) and blood (right panel) of mice after intraperitoneal infection with 1×10^6^ Ba-parental, BaΔ*wadC* or 1×10^5^ *S.* Typhimurium SL1344. (**C**), TNF-α, IL-6, IL-10 and IL-12 p40/p70 levels in the sera of mice infected with Ba-parental or BaΔ*wadC*. Each point is the mean ± standard deviation (n = 5). Differences between Ba-parental and BaΔ*wadC* are indicated with symbols (*, p<0.05; #, p<0.001).

BaΔ*wadC* displayed attenuation in mice (Figure 2A, left panel) with an estimated spleen clearance time of 27 weeks (66 weeks for Ba-parental). This attenuation, however, was less marked than that of a R mutant (BaTn5::*per*) blocked in the synthesis of the only sugar (N-formylperosamine) of the O-PS but with a complete core (Figure 2A, left panel). BaΔ*wadC* induced a transient splenomegaly, in contrast to the increasing splenomegaly observed in Ba-parental and the almost absence of splenomegaly observed in the BaTn5::*per* inoculated mice (Figure 2A, right panel). Complementation with plasmid p*wadC* restored the replication, persistence and splenomegaly of BaΔ*wadC* back to levels observed with the virulent Ba-parental (Figure S5).

Upon infection, BaΔ*wadC* triggered a more intense leukocyte recruitment in the peritoneum and blood than Ba-parental (both at 10^6^ CFU/mouse) but less than the highly endotoxic *Salmonella enterica* subspecies enterica serotype Typhimurium (*S.* Typhimurium) (at 10^5^ CFU/mouse) (Figure 2B). Concurrently, cytokine levels (TNFα, IL-6, IL-12 p40/p70 and IL-10) measured in the serum of BaΔ*wadC* infected mice were always higher than those induced by the Ba-parental (Figure 2C).

### BaΔ*wadC* mutant activates dendritic cells

**Figure 3:**
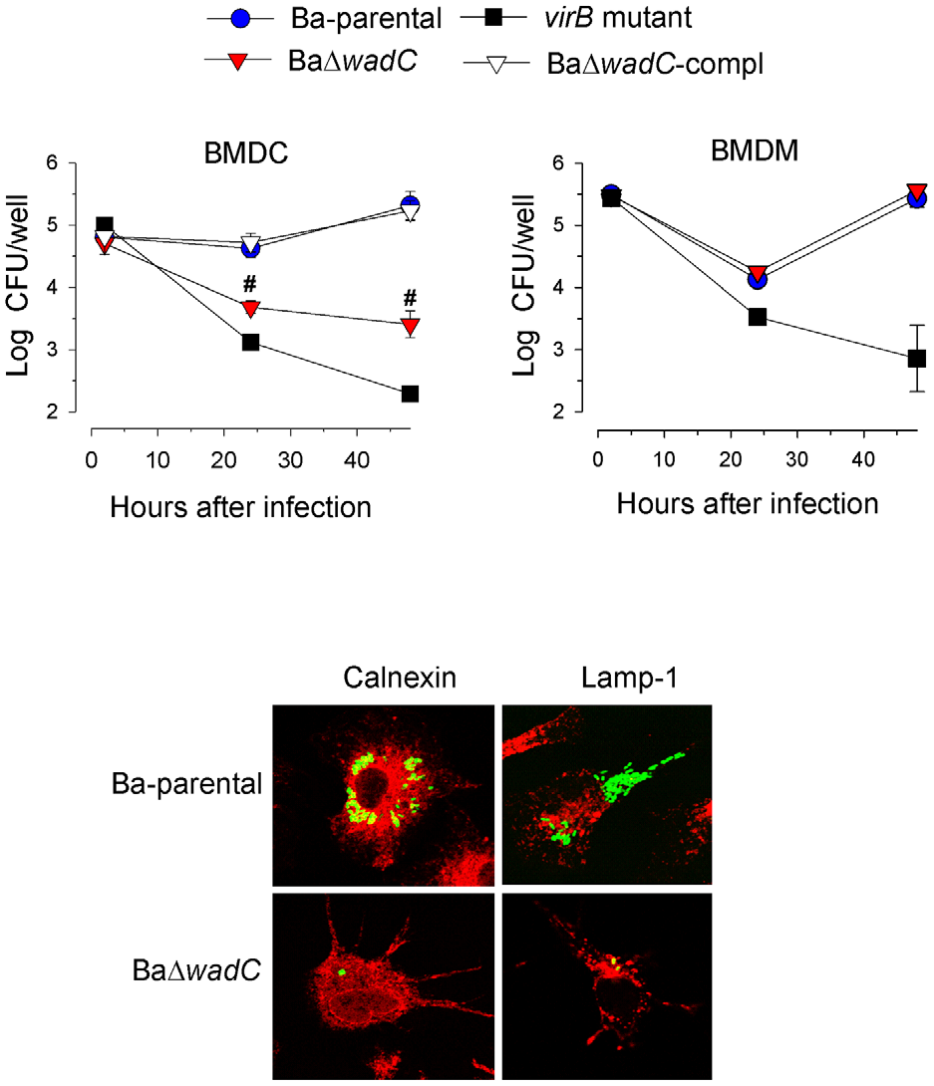
BaΔ*wadC* is attenuated in BMDC. **Upper panel**, intracellular replication of Ba-parental, BaΔ*wadC*, BaΔ*wadC*-compl and *virB* mutant in BMDC and BMDM. Each point is the mean ± standard error of an experiment performed in triplicate, and the results representative of three independent experiments. Differences between Ba-parental and BaΔ*wadC* are indicated with symbols (#, p<0.001). **Lower panel**, confocal imaging of Ba-parental and BaΔ*wadC* carrying a GFP plasmid at 24 h post-infection (calnexin and LAMP-1 are in red).

**Figure 4:**
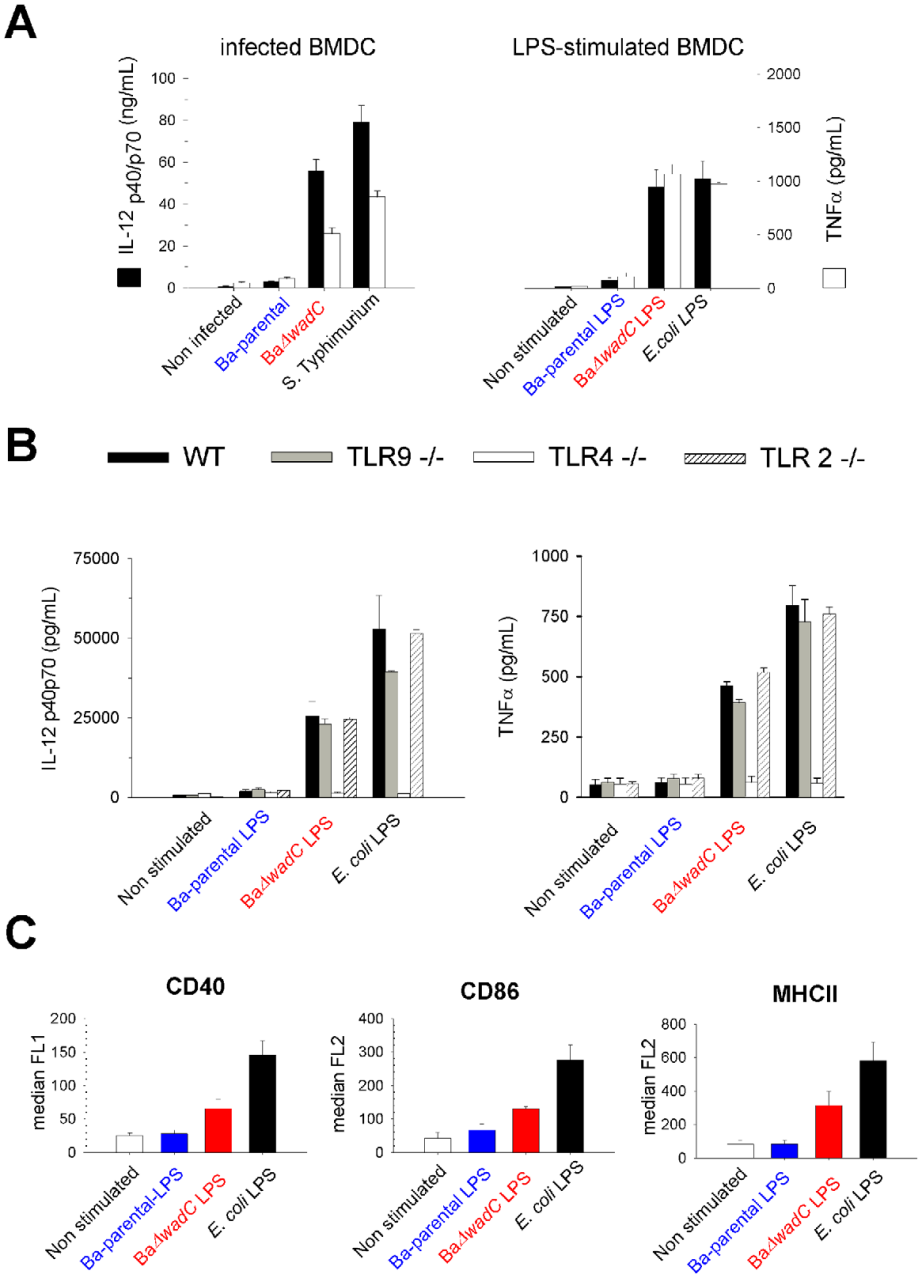
BaΔ*wadC* induces a cytokine release that is reproduced by purified LPS in a TLR-4 dependent fashion. (**A**), IL-12 p40/p70 and TNF-α released by BMDC 24 h after infection (left panel) or stimulation with 10 µg/mL of Ba-parental or BaΔ*wadC* LPS, or with 100 ng/mL of *E. coli* LPS (right panel) measured by ELISA; (**B**), IL-12 p40/p70 (left panel) and TNF-α (right panel) released by BMDC from wild-type (WT), TLR9−/− TLR4−/− or TLR2−/− mice 24 h after stimulation with 10 µg/mL of Ba-parental or BaΔ*wadC* LPS, or with 100 ng/mL of *E. coli* LPS measured by ELISA; (**C**), Surface expression of CD40, CD86 and MHCII in LPS-stimulated BMDC measured by flow cytometry. Each point is the mean ± standard error (n = 3). But for TLR4−/− mice differences between Ba-parental and BaΔ*wadC* or their corresponding LPS were statistically significant (p<0.001). Results are representative of two independent experiments.

We then investigated whether the attenuation and inflammatory responses in mice were reproduced in target cells (dendritic cells and macrophages). BaΔ*wadC* but not the complemented strain BaΔ*wadC*-comp was killed in bone marrow-derived dendritic cells (BMDC), although less rapidly than the virB9 type IV secretion mutant used as a reference of attenuation (Figure 3, upper panel). Moreover, in contrast to the Ba-parental strain, which segregated at 24 h post-infection within the calnexin-positive endoplasmic reticulum, the BaΔ*wadC* mutant-containing vacuoles colocalized with the lysosomal LAMP-1 marker, indicating its failure to reach the endoplasmic reticulum replication niche (Figure 3, lower panel). This attenuation was paralleled by an increased number of Dendritic Cells Aggresome-Like Structures (DALIS) in infected BMDC (Figure S6) indicating that dendritic cells were programmed to undergo a maturation process. Consequently, secretion of IL-12 and TNF-α proinflammatory cytokines in BaΔ*wadC*-infected BMDC at 24 h post-infection reached intermediate levels between those of Ba-parental and *S.* Typhimurium (Figure 4A, left panel). These observations were fully consistent with the results obtained in mice. Interestingly, BaΔ*wadC* multiplied as Ba-parental in bone marrow-derived macrophages (BMDM; Figure 3, upper panel), Raw 264.7 macrophages or HeLa cells (not shown) (see above).

### BaΔ*wadC* mutant is sensitive to complement and bactericidal peptides

**Figure 5:**
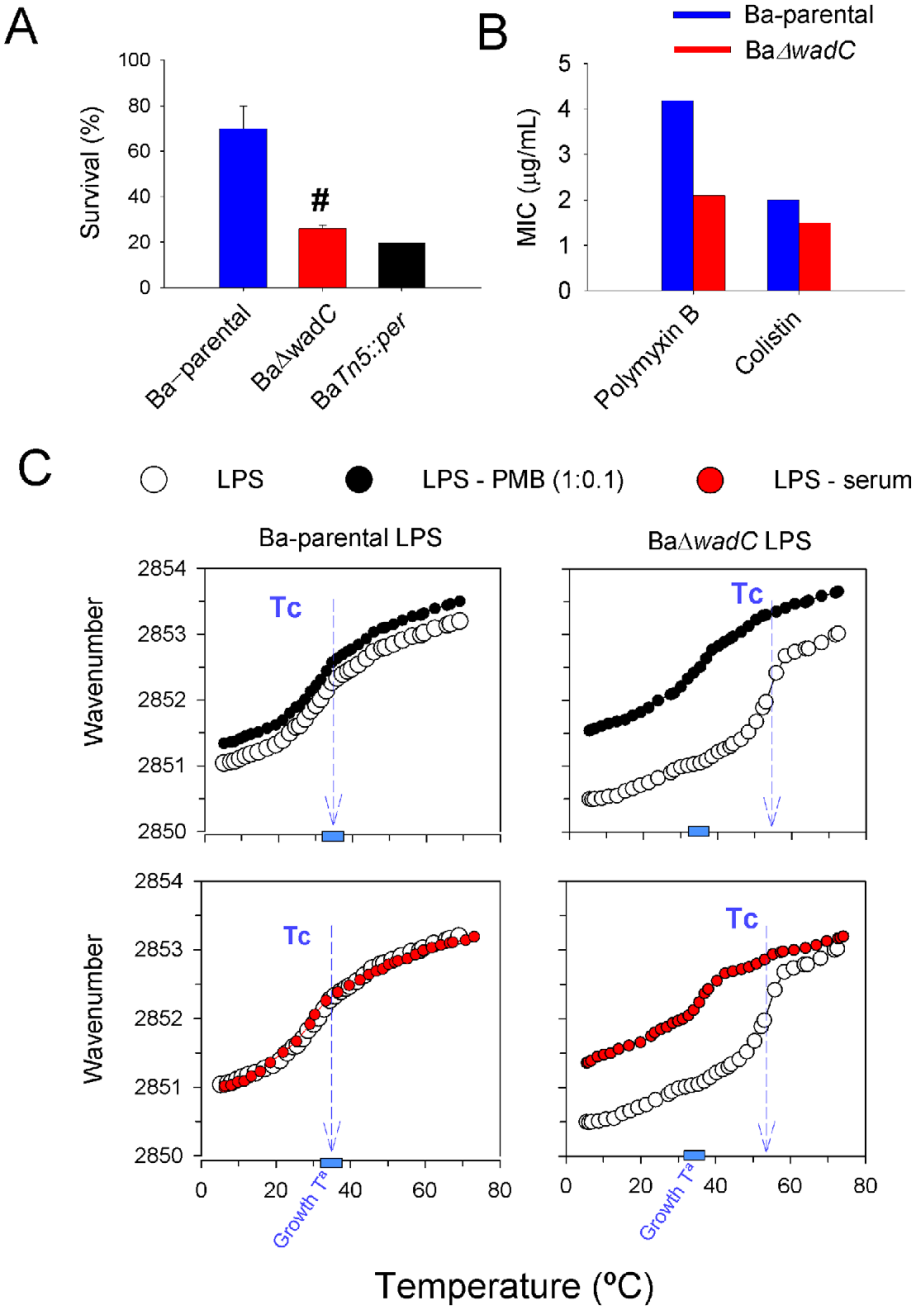
BaΔ*wadC* shows increased sensitivity to normal serum and bactericidal peptides that relates to the LPS core defect. (**A**), Survival of Ba-parental, BaΔ*wadC* and BaTn5::*per* after incubation in non-immune calf serum for 90 min. Each point is the mean ± standard error of an experiment performed in triplicate, and the results representative of five independent experiments (differences between Ba-parental and BaΔ*wadC* are indicated as #, p<0.001). (**B**), minimal inhibitory concentrations (MIC) of polymyxin B (determined by the serial dilution method) and colistin (determined by the E-test) (results representative of three independent experiments). (**C**), Crystalline to fluid (β↔α) phase transition of the hydrocarbon chains of Ba-parental LPS and BaΔ*wadC* LPS measured in the presence (filled circles) or absence (empty circles) of normal human serum (red circles) or polymyxin B (black circles; LPS∶PMB 1∶0.1 molar ratio). The plots represent the position (wavenumber) of the peak of the symmetric stretching vibration of the methylene groups νs(CH2) versus temperature. Tc, transition temperature (results are representative of three independent experiments).

In the absence of antibodies, Smooth *Brucella* cells are poor activators of complement and are thus markedly resistant to the bactericidal action of normal serum, a property that has been attributed to the O-chain [15]. BaΔ*wadC* was more sensitive than Ba-parental to the bactericidal action of serum and comparison with the O-PS defective BaTn5::*per* mutant suggested that the LPS core may be as important as the O-PS (Figure 5A). Brucellae are also resistant to bactericidal polycationic peptides [16], [17], a property linked to a steric hindrance by the O-PS [17] as well as to the low negative charge in the core and lipid A LPS sections, as assessed by physicochemical methods [14]. We tested the sensitivity of BaΔ*wadC* to two potent polycationic lipopeptides, polymyxin B and colistin and demonstrated a greater sensitivity of BaΔ*wadC* (Figure 5B) to these two agents. These results were confirmed using synthetic poly-L-lysine and poly-L-ornithine (not shown).

### BaΔ*wadC* LPS triggers dendritic cell maturation and macrophage activation

We then carried out studies with purified LPSs. In experiments performed to stimulate BMDC with purified LPS, the saturating concentration for BaΔ*wadC* LPS was 10 µg/mL. This is one hundred times more than the corresponding concentration of *E. coli* LPS, a result that illustrates the importance of the expression of an endotoxic lipid A for efficient cell activation. However, at 10 µg/mL, BaΔ*wadC* LPS induced the secretion of IL-12 and TNF-α whereas Ba-parental LPS showed very low levels of cytokine secretion (Figure 4A, right panel) also showing that the presence of the core oligosaccharide in addition to the expression of a long acyl-chain lipid A prevent *Brucella* LPS to show a marked endotoxicity. These results closely matched those obtained in infected BMDC (Figure 4A, left panel). Cytokine secretion was TLR4-dependent since no pro-inflammatory cytokines were detected in BMDC from TLR4 but not TLR2 (Figure 4B), TLR6 (not shown) or TLR9 (Figure 4B) knockout mice stimulated with BaΔ*wadC* LPS. Finally, BMDC showed an intermediate matured phenotype as judged by the expression of CD40 and CD86 co-stimulatory molecules and surface MHCII (Figure 4C) that led to efficient cytokine secretion. Although BaΔ*wadC* multiplied in macrophages (BMDM) (Figure 3, upper panel), its core-defective purified LPS was capable of triggering a cytokine response higher than that of the parental Ba-LPS (Figure S7B). These results suggest that signaling by the mutated LPS during BMDM infection and subsequent cell activation did not occur early enough to prevent the mutant from reaching the replicative niche.

### BaΔ*wadC* LPS binds bactericidal peptides and serum complement

Purified S-LPS forms supramolecular aggregates in which the lipid A acyl chains display a characteristic and temperature-dependent fluidity that increases upon the disturbance of the aggregate caused by binding of bactericidal peptides and complement molecules [18]. Accordingly, we measured this parameter in the absence or the presence of serum or polymyxin B. In the absence of any agent, the β↔α transition that marks the shift from crystalline to fluid phase took place in the 30 to 40°C range for *B. abortus* wild type LPS, with a transition temperature of 37°C (Figure 5C). The LPS of the BaΔ*wadC* mutant showed a very different acyl chain fluidity profile with a transition temperature between 45 and 55°C, and with a markedly more restricted fluidity below transition temperature than the wild type LPS. Therefore, the mutant LPS aggregates were in the crystalline phase at physiological temperatures and, since the acyl-chain composition of lipid A was not significantly affected (Figure S4), we attributed this to the disruption induced in the core structure by mutation of *wadC*. Despite this greater rigidity, the LPS aggregates of the BaΔ*wadC* mutant were clearly affected by normal serum whereas those of Ba-parental LPS were not (Figure 5C). Moreover, when we measured the effect of polymyxin B on acyl chain fluidity, we found a much less marked effect on wild type LPS than on the LPS of the BaΔ*wadC* mutant (Figure 5B). These results show that the core defect is uncovering the complement and polycations targets (Kdo and lipid A phosphate groups) that exist in the innermost sections of LPS [19], and are in agreement with the serum complement and bactericidal peptide sensitivity of the mutant.

### The core of *B. abortus* LPS interacts with MD-2

**Figure 6:**
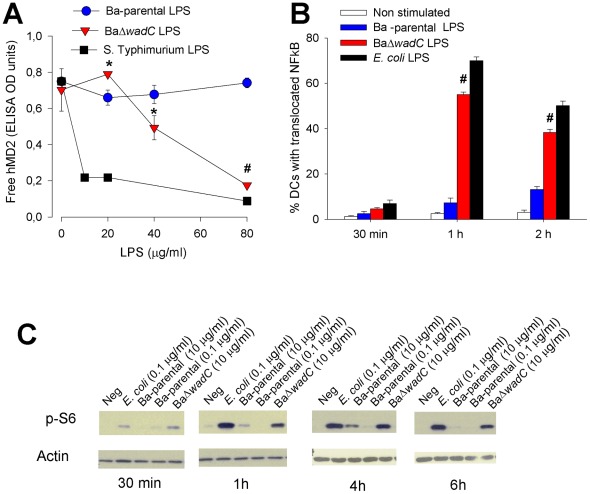
BaΔ*wadC* LPS binds to MD-2 and increases NF-κB translocation and S6 phosphorylation. (**A**) LPS binding to hMD-2. After incubation of 0.75 µM hMD-2 with increasing LPS concentrations, the fraction of hMD-2 not bound to LPS was detected with free-hMD-2 specific antibody 9B4 by ELISA (results are representative of three independent experiments run in triplicate; differences between Ba-parental and BaΔ*wadC* are indicated as *, p<0.05 or #, p<0.001) (**B**) NF-κB translocation to the nucleus. BMDC were stimulated for 1 h with media, *E. coli* LPS (100 ng/mL), Ba-parental LPS (10 µg/mL) or BaΔ*wadC* LPS (10 µg/mL), cells fixed in 3% paraformaldehyde at 37°C for 15 min, immunostained for CD11c and MHCII, and the % of cells showing nuclear translocation of the NF-κB subunit p65/RelA recorded (results are representative of three independent experiments run in triplicate; differences between Ba-parental and BaΔ*wadC* are indicated as #, p<0.001). (**C**) S6 phosphorylation. BMDC were stimulated for 30 min, 1, 4, or 6 h with (left to right) media, *E. coli* LPS (100 ng/mL), Ba-parental LPS (100 ng/mL), Ba-parental LPS (10 µg/mL) or BaΔ*wadC* LPS (10 µg/mL). Cell lysates (30 µg protein) were analyzed by Western blot with a polyclonal antibody against S6-P or an anti-actin antibody as control.

Most endotoxic effects of enterobacterial LPS depend on the interaction with the TLR4 co-receptor MD-2, an event that triggers a cascade of signals leading to the NF-kB-dependent expression of immune response genes and a subsequent proinflammatory response [7]. Therefore, we explored the interaction of BaΔ*wadC* LPS with MD-2 using a competitive ELISA using an antibody that recognizes free- but not LPS-bound human MD-2 (hMD-2) [20]. Whereas Ba-parental LPS did not interact with MD-2 in the range of concentrations tested, BaΔ*wadC* LPS was capable of binding to MD2, although at concentrations higher than the reference endotoxic *Salmonella* LPS (Figure 6A) or *E. coli* LPS (not shown). These results were confirmed by testing the displacement of bis-ANS from MD-2. Whereas Ba-parental LPS did not cause displacement in the range of the concentrations tested (1.25–10 µg/mL), BaΔ*wadC* displaced this probe from MD-2. Consistent with the results of the competitive ELISA, the interaction of the mutated LPS with MD-2 observed using this protocol did not reach the *Salmonella* LPS levels (Figure S8). Interestingly, the LPS of *Ochrobactrum anthropi* (which carries a *Brucella*-type lipid A but differs markedly in the core structure [8], [14]) showed a higher binding to MD2 than either the mutant or the wild-type *B. abortus* LPS (Figure S9). Moreover, the percentage of BMDC showing NF-κB translocation to the nucleus (Figure S10) after 1 h of stimulation with BaΔ*wadC* LPS was clearly above that of Ba-parental LPS (Figure 6B). Clearly, both sets of results are in agreement with the cytokine profiles observed in mice and target cells. In addition, since activation of the mTOR pathway has been implicated in dendritic cell maturation and cytokine production, we determined the phosphorylation of S6, one of the downstream elements of this pathway [21]. When BaΔ*wadC* LPS was compared to Ba-parental LPS, the former induced an earlier, more intense and more sustained S6 phosphorylation (Figure 6C) as detected from 30 min up to 6 h of LPS stimulation.

All this evidence suggests that the core of *B. abortus* LPS negatively modulates recognition by MD-2/TLR4 and the subsequent intracellular signaling leading to pro-inflammatory response and dendritic cell maturation. In support of this interpretation, the TLR4 dysfunction did not affect the multiplication of the wild type but allowed a better replication of the mutant in the spleens of mice (Figure S11). The replication of the *wadC* mutant in TLR4^−^/− did not reach the levels of the wild type, consistent with the existence of additional factors causing attenuation such as the complement and polycation sensitivity observed *in vitro*.

## Discussion

We have identified a *B. abortus* LPS gene (*wadC*) whose disruption does not result in the loss of the O-PS but in an altered core, which is in contrast to all *Brucella* LPS genes described [10], [22], [23], [24]. This allowed us to discriminate the role of the LPS core oligosaccharide from that of the O-polysaccharide in *Brucella* virulence. The *wadC* LPS core mutant induced a strong proinflammatory response and was attenuated in mice and in dendritic cells. These properties were reproduced by the purified *wadC* mutant LPS but not by the wild type Ba-parental LPS. Activation was TLR4-dependent and the core-mutated LPS displayed increased binding to MD-2, the TLR4 co-receptor, which paralleled an increased intracellular signaling. This is the first description of an LPS core hampering complement activation, cationic peptide binding and detection by TLR4-MD-2. Therefore, *B. abortus* LPS core acts as a shield against innate immunity recognition and represents a novel virulence-related mechanism. Studies in progress with the *B. melitensis*, *B. suis* and *B. ovis* mutants in the *wadC* orthologues confirm that this is a general mechanism of this group of bacteria. Indeed, all our results also provide experimental proof that the stealthy behavior of this pathogen towards innate immunity is essential for its virulence, as proposed before [2], [8].

Consistent with the key role of dendritic cells in brucellosis [25], [26], [27], the attenuation and proinflammatory responses of the *wadC* mutant in mice were reproduced in BMDC. It is noteworthy that the attenuation was not observed in macrophages, and this could be attributed to functional differences between these two types of cells in the ability to process and present antigens. Nevertheless, the results obtained in mice showed that the observations made in dendritic cells are more relevant for the course of the infection than those in macrophages *in vitro*. Interference with dendritic cell maturation is a strategy that prevents the development of efficient immunity and there are several examples of intracellular pathogens that target dendritic cells. *Mycobacterium tuberculosis* interferes with TLR signaling in dendritic cells blocking their maturation and IL-12 generation and directing the immunoresponse towards IL-10 production [28]. *S.* Typhimurium, on the other hand, does not block maturation but prevents MHC II antigen presentation in some subtypes of dendritic cells [29], [30]. *Francisella tularensis*, another gram-negative pathogen that does not block dendritic cell maturation, seems to be capable of multiplying in these cells and to inhibit the secretion of pro-inflammatory cytokines [31]. However, only *B. abortus*, *B. melitensis* and *B. suis*, the three classical smooth *Brucella* spp., have been reported to be simultaneously able to multiply in dendritic cells and to prevent their maturation, thereby thwarting the efficient presentation of proteins in either the MHC I or MHC II context [27]. The properties that enable the brucellae to display these abilities are beginning to be understood. TLR2, TLR4 and TLR9 seem to be the most important TLRs in dendritic cells, and they recognize lipoproteins, LPS and CpG DNA, respectively. Salcedo et al. [27] have recently shown that protein Btp1 of *B. abortus* interferes with the TLR2 signaling pathway and down modulates dendritic cell activation. Billard et al. [26] found that the response of dendritic cells derived from peripheral blood monocytes to wild type *B. abortus* LPS was low as compared to that obtained with *E. coli* LPS. Our results extend these observations and demonstrate the connection between the LPS core oligosaccharide and the ability of *B. abortus* to thrive in dendritic cells and to circumvent their maturation.

Chemical analyses of the *B. abortus* LPS core reveal the presence of Kdo, glucosamine, glucose, mannose and quinovosamine [6]. Our results suggest that these sugars must be arranged in such a way that some of them probably play no role in the section linking the O-PS, as predicted before by studies with monoclonal antibodies [23] and on the inability of polymyxin B to neutralize the charge of *B. abortus* LPS [14]. Moreover, we have identified a second glycosyltransferase gene (*wadB*) that, upon disruption, generates an LPS phenotype close to the one described here for BaΔ*wadC*. These genetic data indicate that at least two core sugars must be arranged in a structure whose damage does not affect the link to the O-PS. Research in progress shows the existence of a mannose containing branch in the *Brucella* LPS core. WadC is a putative mannosyltransferase and it seems likely that it could be involved in the transfer of a mannose unit to such a branch. It is tempting to speculate that such a structure hinders the access to the Kdo and lipid A phosphates targeted by bactericidal peptides as well as complement C1q [19], and could thus account for the results of the transition measurements performed in the presence of PMB and serum. We also propose that the full core structure is one of the factors contributing to a defective MD-2 recognition, the other one being the peculiar acyl chain composition of *Brucella* lipid A. Two not mutually exclusive hypotheses could account for the role of the complete core. Its absence in the mutant could favor the dissociation of individual molecules from aggregates and make them more readily available for binding to MD2, and the anionic molecules and Kdo in the inner sections could be more accessible for binding to MD2. In addition, it is known that C12-C14 hexaacylated lipids A like that of *E. coli* interact with a large hydrophobic groove in MD-2, with five acyl chains deep inside, the remaining chain in a hydrophobic interaction with TLR4 and the bisphosphorylated glucosamine disaccharide tilted outwards [7]. Therefore, the lipid A phosphate groups contribute to receptor multimerization by interacting with positively charged residues in TLR4 and MD-2 [7]. Previously, we proposed that the very long chain fatty acids in *Brucella* lipid A are critical in preventing effective recognition by TLR4-MD-2 [32] and here we show that the interaction of both the wild type and the BaΔ*wadC* LPS with MD-2 is significantly reduced as compared to that of *Salmonella* LPS. This suggests that the MD-2 hydrophobic pocket does not allow for efficient interaction with the bulky lipid A of *Brucella*. Moreover, removal of part of the *B. abortus* LPS core increases the binding to MD-2 indicating that interaction is hampered by virulent *B. abortus* intact core. Since *O. anthropi* and *B. abortus* LPS have similar a lipid A but a markedly different core structure [8], [14], the results obtained with the former in the MD2 assay also suggest that core structures are important. The work in progress on the structure of the *B. abortus* LPS core shows that the O-PS stems from a few sugars linked to Kdo I, suggesting Kdo II as the section linked to the sugar(s) removed by the wadC mutation. Thus, these sugars should be close to the negatively charged groups in the lipid A backbone and Kdo and could hinder cationic peptide, C1q and MD-2 interactions. This structure may not be unique to *B. abortus*. In fact, *wadC* homologues are found not only in the genomes of all *Brucella* species but also in *Bartonella* spp., suggesting structures acting as shields against innate immunity recognition.

Finally, current classical smooth brucellosis vaccines (*B. abortus* S19 and *B. melitensis* Rev 1) are doubtlessly useful tools in animal vaccination and, therefore, in the eradication of this zoonotic disease, but do not afford 100% protection in the natural host. Hence, their successful use requires complementary measures (tagging and control of animal movement, efficient veterinary services, repeated animal testing) that make brucellosis eradication a cumbersome and long process [33]. We found that the cytokine profile, with IL-12 being released in large amounts, the transitory splenomegaly and the eventual clearance of BaΔ*wadC* could make mutation of *wadC* a tool to improve existing or future vaccines.

## Materials and Methods

### Ethics

Animal experimentation was conducted in strict accordance with good animal practice as defined by the French animal welfare bodies (Law 87–848 dated 19 October 1987 modified by Decree 2001-464 and Decree 2001-131 relative to European Convention, EEC Directive 86/609). All animal work was approved by the Direction Départmentale des Services Vétérinaires des Bouches du Rhônes (authorization number 13.118).

All animals were handled and sacrificed according to the approval and guidelines established by the “Comité Institucional para el Cuido y Uso de los Animales” of the Universidad de Costa Rica, and in agreement with the corresponding law “Ley de Bienestar de los Animales No 7451” of Costa Rica (http://www.micit.go.cr/index.php/docman/doc_details/101-ley-no-7451-leyde-bienestar-de-los-animales.html).

Mice (Charles River, Elbeuf, France) were accommodated in the animal building of the CITA of Aragón (ID registration number ES-502970012005) in cages with water and food ad libitum and under biosafety containment conditions, for 2 weeks before the start and all along the experiment. The animal handling and procedures were in accordance with the current European legislation (directive 86/609/EEC) supervised by the Animal Welfare Committee of the institution (protocol numberR102/2007).

### Bacterial strains and growth conditions

The bacterial strains and plasmids used are listed in Table S1. Moreover, the strain *Salmonella enterica* subespecies *enterica* serotype *typhimurium* (abbreviated as *S.* Typhimurium) reference ATCC SL1344, *Salmonella abortus equi* strain HL83 and *E. coli* strain MG1655 were used as controls in some assays. Bacteria were routinely grown in standard tryptic soy broth or agar either plain or supplemented with kanamycin at 50 µg/mL, or/and nalidixic acid at 25 µg/mL, or/and 5% sucrose. All strains were stored in skim milk at −80°C.

### DNA manipulations

Plasmid and chromosomal DNA were extracted with Qiaprep spin Miniprep (Qiagen GmbH, Hilden, Germany), and Ultraclean Microbial DNA Isolation kit (Mo Bio Laboratories) respectively. When needed, DNA was purified from agarose gels using Qiack Gel extraction kit (Qiagen) and sequenced by the Servicio de Secuenciación de CIMA (Centro de Investigación Médica Aplicada, Pamplona, Spain). Primers were synthesized by Sigma-Genosys Ltd. (Haverhill, United Kingdom). Searches for DNA and protein homologies were carried out using the NCBI (National Center for Biotechnology Information; http://www.ncbi.nlm.nih.gov) and the EMBL-European Bioinformatics Institute server (http://www.ebi.ac.UK/ebi_home.html). In addition, sequence data were obtained from The Institute for Genomic Research website at http://www.tigr.org. Genomic sequences of *B. melitensis*, *B. abortus* and *B. suis* were analyzed using the database of the URBM bioinformatic group (http://urbm-cluster.urbm.fundp.ac.be/~apage).

### Construction of the Ba-parental *wadC* non polar mutant (BaΔ*wadC*)

In-frame deletion mutant BaΔ*wadC* was constructed by PCR overlap using genomic DNA of Ba-parental as DNA template (Figure S1). Primers were designed based on the available sequence of the corresponding genes in *B. abortus* 2308. For the construction of the *wadC* mutant, we first generated two PCR fragments: oligonucleotides *wad*C-F1 (5′-CTGGCGTCAGCAATCAGAG-3′) and *wadC*-R2 (5′- GTGCAACGACCTCAACTTCC-3′) were used to amplified a 476-bp fragment including codons 1 to 16 of the *wadC* ORF, as well as 424 bp upstream of the *wad*C start codon, and oligonucleotides *wadC*-F3 (5′-GGAAGTTGAGGTCGTTGCACACGCCATC GAACCTTATCTG-3′) and *wadC*-R4 (5′-CGGCTATCGTGCGATTCT-3′) were used to amplify a 453-bp fragment including codons 308 to 354 of the *wadC* ORF and 320-bp downstream of the *wadC* stop codon (see S-1). Both fragments were ligated by overlapping PCR using oligonucleotides *wadC*-F1 and *wadC*-R4 for amplification, and the complementary regions between *wadC*-R2 and *wadC*-F3 for overlapping. The resulting fragment, containing the *wadC* deletion allele, was cloned into pCR2.1 (Invitrogen), to generate plasmid pRCI-23, sequenced to ensure the maintenance of the reading frame, and subsequently subcloned into the BamHI and the XbaI sites of the suicide plasmid pJQK. The resulting mutator plasmid (pRCI-26) was introduced in Ba-parental by conjugation. The first recombination (integration of the suicide vector in the chromosome) was selected by Nal and Kan resistance, and the second recombination (excision of the mutator plasmid leading to construction of the mutant by allelic exchange), was selected by Nal and sucrose resistance and Kan sensitivity. The resulting colonies were screened by PCR with primers *wadC*-F1 and *wadC*-R4 which amplify a fragment of 929 bp in the mutant and a fragment of 1805 bp in the parental strain. The mutation generated results in the loss of the 82% of the wadC ORF, and the mutant strain was called BaΔ*wadC*.

### Complementation of Ba*ΔwadC*

Taking into account that the WadC sequences of *B. melitensis* and *B. abortus* are identical, we used the *B. melitensis* ORFeome constructed with the *Gateway cloning Technology* (Invitrogen) for complementation [34]. The clone carrying *B. melitensis wadC* was extracted, and the DNA containing the corresponding ORF was subcloned in pRH001 [35] to produce plasmid p*wadC*. To complement the *wadC* mutation, plasmid p*wadC* was introduced into the Ba*ΔwadC* mutant by mating with *E. coli* S17-1 and the conjugants harboring p*wadC* (designated as *BaΔwadC*-comp) were selected by plating the mating mixture onto TSA-Nal-Kan plates which were incubated at 37°C for 3 days.

### Virulence assay in mice

Seven-week-old female BALB/c mice (Charles River, Elbeuf, France) were kept in cages with water and food ad libitum and accommodated under biosafety containment conditions 2 weeks before the start of the experiments. Inocula were prepared in sterile 10 mM PBS (pH 6.85). For each strain, 30 mice were inoculated intraperitoneally with 0.1 mL of inoculum containing 5.8×10^4^ (Ba-parental) or 4.9×10^4^ (Ba*ΔwadC*) CFU/mouse and the number of CFU in spleens (n = 5) was determined at 1, 2, 4, 6, 8, and 12 weeks after inoculation. BaTn5::*per* was used as control of representative R mutant with complete LPS-core, at inoculation dose of 1×10^8^ CFU intraperitoneally and viable counts in spleens at 1, 2, 3, 6 and 9 weeks post-infection. An additional experiment was performed under the same conditions but including Ba*ΔwadC*-comp and the number of CFU in spleens was determined 8 weeks after inoculation. The identity of the spleen isolates was confirmed by PCR at each time-point during the infection process. The individual data were normalized by logarithmic transformation, and the mean and standard deviation (SD) of log_10_ CFU/spleen were calculated.

### Leukocyte counts

BALB/c mice from 20 and 24 g were intraperitoneally injected with 10^6^ CFU of Ba-parental, 10^5^ of *S.* Typhimurium or 0.1 mL pyrogen-free sterile PBS. Blood was collected from the retro-orbital sinus and subjected to analysis. Alternatively, 5 mL of ice cold PBS were injected in the peritoneal cavity of killed the mice, and the fluids collected with a syringe (from 3.8 to 4.5 mL) from exposed peritoneal cavity. Then fluids were centrifuged and the peritoneal cells resuspended in 0.2 mL of PBS and counted in Neubauer chambers. Giemsa-Wright staining smears were performed to distinguish between leukocytes.

### Intracellular multiplication

Bone marrow cells were isolated from femurs of 7–8-week-old C57Bl/6 female, TLR2−/−, TLR4−/− or TLR9−/− [36], [37] mice and differentiated into either dendritic cells (BMDCs) or macrophages (BMDMs) as described previously [38], [39] in presence of decomplemented fetal bovine serum. Infections were performed by centrifuging the bacteria onto the differentiated cells (400×*g* for 10 min at 4°C; bacteria: cells ratio of 20∶1 for BMDCs or 50∶1 for BMDMs) followed by incubation at 37°C for either 15 min (BMDMs) or 30 min (BMDCs) under a 5% CO_2_ atmosphere. Cells were either extensively washed (BMDMs) or gently washed (BMDCs) with medium to remove extracellular bacteria and incubated in medium supplemented with 100 µg/mL gentamicin for 1 h to kill extracellular bacteria. Thereafter, the antibiotic concentration was decreased to 20 µg/mL. To monitor *Brucella* intracellular survival, infected cells were lysed with 0.1% (vol/vol) Triton X-100 in H_2_O (BMDCs) or after PBS washing (BMDMs) and serial dilutions of lysates were rapidly plated onto tryptic soy agar plates to enumerate CFU.

### Cytokine measurement

The levels of TNF- α, IL-6, IL-10 and IL-12 p40/p70 were estimated at different time points by enzyme-linked immunosorbent assays (ELISA) in the sera of BALB/c mice infected intraperitoneally, and in the *s*upernatants of BMDC or BMDM at 24 hours after infection (see above) or after stimulation with 10 µg/mL of the appropriate LPS from different *Brucella* strains or 100 ng/mL from *E. coli* ATCC 35218 obtained by the phenol-water procedure and purified further by the phenol-water-deoxycholate method [40]. For the latter purpose, a stock of 1 mg/mL in pyrogen free sterile water was prepared, sonicated briefly and sterilized by autoclaving. Prior to use, the stock was heated at 56°C for 15 min and then cooled to room temperature.

### Immunofluorescence assays

BMDCs were grown on glass coverslips and inoculated with bacteria as described above or stimulated with the appropriate LPS. At different times after inoculation (see Results), coverslips were fixed with 3% paraformaldehyde pH 7.4 at 37°C for 15 min and washed three times with PBS. Coverslips were processed for immunofluorescence staining as previously described [39]. Briefly, cells were permeabilized with 0.1% saponin and incubated with primary antibodies. After several washes, the primary antibodies were revealed with the appropriate secondary antibodies. The primary antibodies used for immunofluorescence microscopy were: cow anti-*B. abortus*; rat anti-mouse LAMP1 ID4B (Developmental Studies Hybridoma Bank, National Institute of Child Health and Human Development, University of Iowa); mouse anti FK2 (Biomol); Moab anti-calnexin (kindly provided by Dr. D. Williams, University of Toronto) and NF-κB subunit p65/RelA (Santa Cruz). In all experiments, BMDCs were labeled using an antibody against a conserved cytoplasmic epitope found on MHC-II I-A ß subunits or MHC II [29] which does not produce significant labeling with BMDMs. In addition, BMDCs were labeled with an anti–CD11c antibody (Biolegend) confirming that they are dendritic cells [27]. Samples were analyzed under a Leica DMRBE epifluorescence microscope for quantitative analysis, or a Zeiss LSM 510 laser scanning confocal microscope for image acquisition. Images were then assembled using Adobe Photoshop 7.0. Quantifications were done by counting at least 300 cells in 3 independent experiments.

### Immunoblotting

30 µg of cell lysates were subjected to SDS-PAGE and, after transfer to nitrocellulose, the membrane was probed with a polyclonal antibody against phospho-S6 (Cell Signaling Technology) that detects phosphorylation on Ser235/236 or anti-actin antibody. Blots were subjected to enhanced chemiluminescence detection (ECL, PIERCE).

### Flow cytometry

BMDCs treated with different types of LPS's were collected and stained immediately before fixation with paraformaldehyde. Isotype controls were included as well as BMDCs treated with the different secondary antibodies for control of autofluorescence. Cells were always gated on CD11c and a minimum of 12,000 CD11c-positive events were obtained for analysis. A FACScalibur cytometer (Beckton Dickinson) was used and data were analysed using FlowJo software (Tree Star). Allophycocyanin (APC) conjugated-anti-CD11c antibody (HL3) from Pharmigen was used in all experiments along with either phycoerythrin (PE) conjugated anti-CD86, anti-IA-IE (MHC class II) or fluorescein isothiocyanate (FITC) conjugated anti-CD40, all from BioLegend.

### LPS extraction and characterization

Extraction of whole-cell LPS by SDS-proteinase K protocol was performed as described previously [41]. In addition, LPS was obtained by methanol precipitation of the phenol phase of a phenol-water extract [13]. This fraction (10 mg/mL in 175 mM NaCl, 0.05% NaN_3_, 0.1 M Tris-HCl [pH 7.0]) was then purified by digestion with nucleases (50 µg/mL each of DNase-II type V, and RNase-A [Sigma, St. Louis, Missouri, U.S.A.], 30 min at 37°C) and three times with proteinase K (50 µg/mL, 3 hours at 55°C), and ultracentrifuged (6 h, 100,000× g). When we applied this method to BaΔ*wadC*, we found that the supernatant of the ultracentrifugation step contained the major fraction of the LPS. The studies were performed with the major LPS fractions of each bacteria. Free lipids (ornithine lipids and phospholipids) were then removed by a fourfold extraction with chloroform-methanol. (2∶1 [vol/vol]) [14].

LPS were analyzed in 15 or 18% polyacrylamide gels (37.5∶1 acrylamide/methylene-bisacrylamide ratio) in Tris-HCl-glycine and stained by the periodate-alkaline silver method [42]. For Western blots, gels were electrotransferred onto nitrocellulose sheets (Schleicher & Schuell GmbH, Dassel, Germany), blocked with 3% skim milk in PBS with 0.05% Tween 20 overnight, and washed with PBS–0.05% Tween 20. Immune sera were diluted in this same buffer and, after incubation overnight at room temperature, the membranes were washed again. Bound immunoglobulins were detected with peroxidase-conjugated goat anti-mouse immunoglobulin (Nordic,Tilburg, Netherlands) and 4-chloro-1-naphthol- H_2_O_2_. Monoclonal antibodies (Moabs) used in this study were Cby-33H8 (Ingenasa, Madrid, Spain), which recognizes the C/Y O-chain epitope, and A68/24D08/G09, A68/24G12/A08, and A68/3F03/D5 which recognize core epitopes [43]. The inner core LPS marker Kdo was determined colorimetrically by the thiobarbituric acid method using pure Kdo and deoxyribose as the standards, with the modifications described previously [44]. Kdo contents were 1.6 and 2.4% for the wild type and the mutated LPS, respectively.

### Lipid A analysis

Lipid A fractions were extracted using an ammonium hydroxide/isobutyric acid method and subjected to negative ion matrix-assisted laser desorption ionization time-of-flight (MALDI-TOF) mass spectrometry analysis [45], [46]. Briefly, lyophilized crude cells (20 mg) were resuspended in 400 µL isobutyric acid/1M ammonium hydroxide (5∶3, v/v) and were incubated in a screw-cap test tube at 100°C for 2 h, with occasional vortexing. Samples were cooled in ice water and centrifuged (2,000×g for 15 min). The supernatant was transferred to a new tube, diluted with an equal volume of water, and lyophilized. The sample was then washed twice with 400 µL methanol and centrifuged (2,000×g for 15 min). The insoluble lipid A was solubilized in 100 µL chloroform/methanol/water (3∶1.5∶0.25, v/v/v). Analyses were performed on a Bruker Autoflex II MALDI-TOF mass spectrometer (Bruker Daltonics, Incorporated) in negative reflective mode with delayed extraction. Each spectrum was an average of 300 shots. The ion-accelerating voltage was set at 20 kV. Dihydroxybenzoic acid (Sigma Chemical Co., St.Louis, MO) was used as a matrix. A few microliters of lipid A suspension (1 mg/mL) was desalted with a few grains of ion-exchange resin (Dowex 50W-X8; H+) in an Eppendorf tube. A 1 µL aliquot of the suspension (50–100 µL) was deposited on the target and covered with the same amount of the matrix suspended at 10 mg/mL in a 0.1 M solution of citric acid. Different ratios between the samples and dihydroxybenzoic acid were used when necessary. A peptide calibration standard (Bruker Daltonics) was used to calibrate the MALDI-TOF. Further calibration for lipid A analysis was performed externally using lipid A extracted from *E. coli* strain MG1655 grown in LB at 37°C.

### LPS binding to hMD-2

Binding of LPS to MD-2 was assayed by two different methods: binding to hMD-2 by competitive ELISA and displacement of bis-ANS (4,4′-Dianilino-1,1′-binaphthyl-5,5′-disulfonic acid dipotassium salt) from MD-2 by LPS. In both assays, LPS was sonicated before use and subjected to three cycles of heating at 56°C followed by cooling to 4°C.

The ELISA for determination of LPS binding to hMD-2 was performed in 96-well plates (NUNC immunoplate F96 cert. Maxi-sorp, Roskilde, Denmark).Chicken anti-hMD-2 (GenTel, Madison, WI, U.S.A) (5 µg/mL) in 50 mM Na_2_CO_3_ (pH 9.6) was used to coat the microtiter plate at 4°C overnight. Excess binding sites were blocked with 1% BSA in PBS buffer (pH 7.2) for 1 h at room temperature, and rinsed three times with the same buffer. During the blocking step, hMD-2 (0.75 µM) was preincubated with 0 µM to 8 µM LPS at 37°C and, as a negative control, LPS was also preincubated in absence of hMD-2. This preincubated solutions were added to the plate, which was then incubated for 1 h at 37°C. After rinsing, hMD-2 not bound to LPS was detected by incubation with 0.1 µg/mL of mouse anti h-MD-2 (clone 9B4 e-Bioscience San Diego, CA., U.S.A.) in PBS buffer at 37°C for 1 h, followed by incubation with 0.1 µg/mL peroxidase-conjugated goat anti-mouse IgG (Santa Cruz, CA., U.S.A.), also in PBS buffer at 37°C for 1 h. After plate washing, ABTS (Sigma) was added, the reaction was stopped with 1% SDS after 15 min, and the absorbance at 420 nm measured using a Mithras LB940 apparatus (Berthold Technologies). *Salmonella abortus equi* (strainHL83) LPS, used as a control, was prepared by a phenol extraction procedure and was kindly provided by Dr. Brandenburg (Forschungszentrum Borstel, Germany).

Since the binding site of bis-ANS overlaps with the MD-2 binding site of LPS we measured the displacement of the probe by the LPS of Ba-parental and BaΔwadC using *Salmonella* LPS as a control [47].Binding of bis-ANS to hMD-2 was measured at 20°C using excitation at 385 nm and measuring the emission fluorescence spectra between 420 and 550 nm. Then, increasing amounts of LPS were added to preincubated bis-ANS/hMD-2 complex (200 nM/200 nM). The F_0_ value was the fluorescence intensity of bis-ANS/hMD-2 complexes at 490 nm after 30 min of incubation (to reach stable fluorescence).The F_LPS_ value was the fluorescence at 490 nm after LPS addition to the complex. Fluorescence was measured on Perkin Elmer fluorimeter LS 55. Quartz glass cuvette (5×5 mm optical path, Hellma Suprasil) was used and bis-ANS was obtained from Sigma (St. Louis, Missouri, U.S.A.).

### LPS-Complement and -polymyxin B interactions determined by acyl-chain fluidity measurements

The transition of the acyl chains of LPS from a well-ordered state (gel phase) to a fluid state (liquid crystalline phase) at a lipid-specific temperature (Tc) was determined by Fourier transform infrared spectroscopy. A specific vibrational band, the symmetric stretching vibration of the methylene groups νs(CH2) around 2,850 cm−1, was analyzed since its peak position is a measure of the state of order (fluidity) of the acyl chains. Measurements were performed in a Bruker IFS 55 apparatus (Bruker, Karlsruhe, Germany) as described previously [48]. To ensure homogeneity, LPS suspensions were prepared in 2.5 mM HEPES (pH 7.2) at room temperature, incubated at 56°C for 15 min, stirred, and cooled to 4°C. This heating/cooling step was repeated three times, and the suspensions were stored at 4°C for several hours before analysis. LPS suspensions (water content, 90%) were analyzed in CaF2 cuvettes with 12.5-µm Teflon spacers, and for each measurement, 50 interferograms were accumulated, Fourier transformed, and converted to absorbance spectra. The measurements were obtained in continuous heating scans (2°C/min) between 10°C and 60°C. To test the effect of complement, the experiments were performed in the presence of normal human serum. The effect of polymyxin B was assessed similarly at different LPS∶polymyxin B molar ratios (see Results), and using an average MW of 11800 for Ba-2308 LPS (determined by SDS-PAGE with *Yersinia enterocolitica* O:8 LPS as a standard).

### Sensitivity to brucellaphages, dyes, antibiotics and polycationic bactericidal peptides

The minimal inhibitory concentrations (MIC) of polymyxin B, poly-L-ornithine, poly-L-lysine, colistin, penicillin, doxycycline, clarithromycin, erythromycin, rifampicin, basic fuchsin, safranine and thionin was determined in Müller-Hinton medium by standard procedures. Sensitivity to the S (Tb, Wb, Iz) and rough (R/C) -specific brucellaphages was measured by testing the lysis of bacteria exposed to serial 10-fold dilutions made from a routine test dilution phage stock [49].

### Sensitivity to the bactericidal action of non-immune serum

Exponentially growing bacteria were adjusted to 10^4^ CFU/mL in saline and dispensed in triplicate in microtiter plates (45 µL per well) containing fresh normal bovine serum (90 µL/well). After 90 min of incubation at 37°C, brain heart infusion broth was dispensed (200 µL/well), mixed with the bacterial suspension and 100 µL was plated on TSA. Results were expressed as the percentage of the average CFU with respect to the inoculum.

### Statistical analysis

The Kolmogorov-Smirnov test was applied to assess the normal distribution of data obtained in each experiment. Thereafter, means were statistically compared by an unpaired Student's t test. Kruskal-Wallis and Mann-Whitney tests were used for experiments with non-normal data distribution. The StatviewGraphics 5.0 for Windows (SAS Institute Inc) statistical package was used in all cases.

## Supporting Information

Figure S1 **BAB1_1522 (BaΔ** ***wadC*** **) and its upstream and downstream regions.** The DNA sequence is registered at NCBI with accession number YP_414888.1 (Gene ID: 3788779). Start and stop codons are in blue characters; grey characters denote intergenic nucleotides; primers used for mutagenesis are in bold characters; red and green characters mark amino acids deleted and present in BaΔ*wadC*, respectively.
(TIF)

Figure S2 **Representative growth curves of Ba-parental and BaΔ** ***wadC*** **in TSB at 37°C.** (each point represents the mean of triplicate samples; the experiment was repeated three times with similar results).
(TIF)

Figure S3 **Ba** ***ΔwadC*** **carries a partially defective LPS core oligosaccharide.** Western blot analyses were performed with monoclonal antibodies Cby-33H8 (O-polysaccharide C/Y epitope) A68/24D08/G09, A68/24G12/A08 and A68/3F03/D5 (core oligosaccharide) and SDS-proteinase K LPS extracts of Ba-parental, BaΔ*wadC* and BaΔ*wadC*-compl (complemented mutant).
(TIF)

Figure S4 **MALDI-TOF analysis of lipid A.** The table summarizes the results obtained with several independent preparations, and the figures below show representative spectra (peak B is an uncharacterized molecular species).
(TIF)

Figure S5 **Complementation with p** ***wadC*** **restores the ability of BaΔ** ***wadC*** **to multiply in mice.** Mice were inoculated intraperitoneally with 5×10^4^ Ba-parental, BaΔ*wadC* and BaΔ*wadC*-compl (complemented mutant). Number of CFU in spleens was determined eight weeks after inoculation. Differences between BaΔ*wadC* and Ba-parental or BaΔ*wadC*-compl were statistically significant. (#, p<0.001).
(TIF)

Figure S6 **BaΔ** ***wadC*** **induces DALIS in BMDC.** (A), Percentages of BMDCs infected with either Ba-parental, BaΔ*wadC* or *S.* Typhimurium that contain DALIS. (B), representative confocal images of BMDCs infected with Ba-parental GFP or BaΔ*wadC* GFP (in green) labeled with Moabs to CD11c (in blue) and FK2 (in red) 24 hours after infection.
(TIF)

Figure S7 **BaΔ** ***wadC*** **LPS induces cytokine release in BMDM.** (A), Intracellular replication of Ba-parental, BaΔ*wadC*, BaΔ*wadC*-compl and virB mutant in BMDM. (B), TNF-α (left panel) and IL-12 p40/p70 (right panel) released by BMDM 24 h after incubation with 10 µg/mL of Ba-parental or BaΔ*wadC* LPS, or with 100 ng/mL of *E. coli* LPS as measured by ELISA.
(TIF)

Figure S8 **LPS binding to MD-2 assessed by displacement of bis-ANS.** The bis-ANS/hMD2 complex (200 nM/200 nM) was incubated for 30 min to reach stable fluorescence (F_0_). Then, increasing amounts of the indicated LPSs were added and fluorescence (F_LPS_) measured. *Salmonella* LPS was used as control. The results shown are representative of three independent experiments.
(TIF)

Figure S9 ***Ochrobactrum anthropi*** **LPS binding to MD-2.** (A), After incubation of 0.75 µM hMD-2 with increasing LPS concentrations, the fraction of hMD-2 not bound to LPS was detected with free-hMD-2 specific antibody 9B4 by ELISA. (B), MALDI-TOF analysis of *Ochrobactrum anthropi* lipid A.
(TIF)

Figure S10 **Nuclear translocation of NF-κB subunit in LPS-stimulated BMDC.** Cells were stimulated for 1 h with cell culture medium, *E. coli* LPS (100 ng/mL), Ba-parental LPS (10 µg/mL) or BaΔ*wadC* LPS (10 µg/mL). Cells were stained with CD11c (in blue) and MHCII (in green). The nuclear translocation NF-κB subunit p65/ReiA (in red) was analyzed by confocal microscopy.
(TIF)

Figure S11 **Multiplication of Ba-parental or BaΔ** ***wadC*** **in the spleens of TLR4 KO mice.** Mice were intraperitoneally inoculated with 1×10^6^ CFU of either Ba-parental or BaΔ*wadC*. Ten weeks after infection the number of CFU per spleen were determined.
(TIF)
