# Supplementary figures and images for: The Lipopolysaccharide Core of Brucella abortus Acts as a Shield Against Innate Immunity Recognition
Source: PLoS Pathog. 2012 May 10;8(5):e1002675. doi: 10.1371/journal.ppat.1002675 (PMC3349745; doi:10.1371/journal.ppat.1002675)

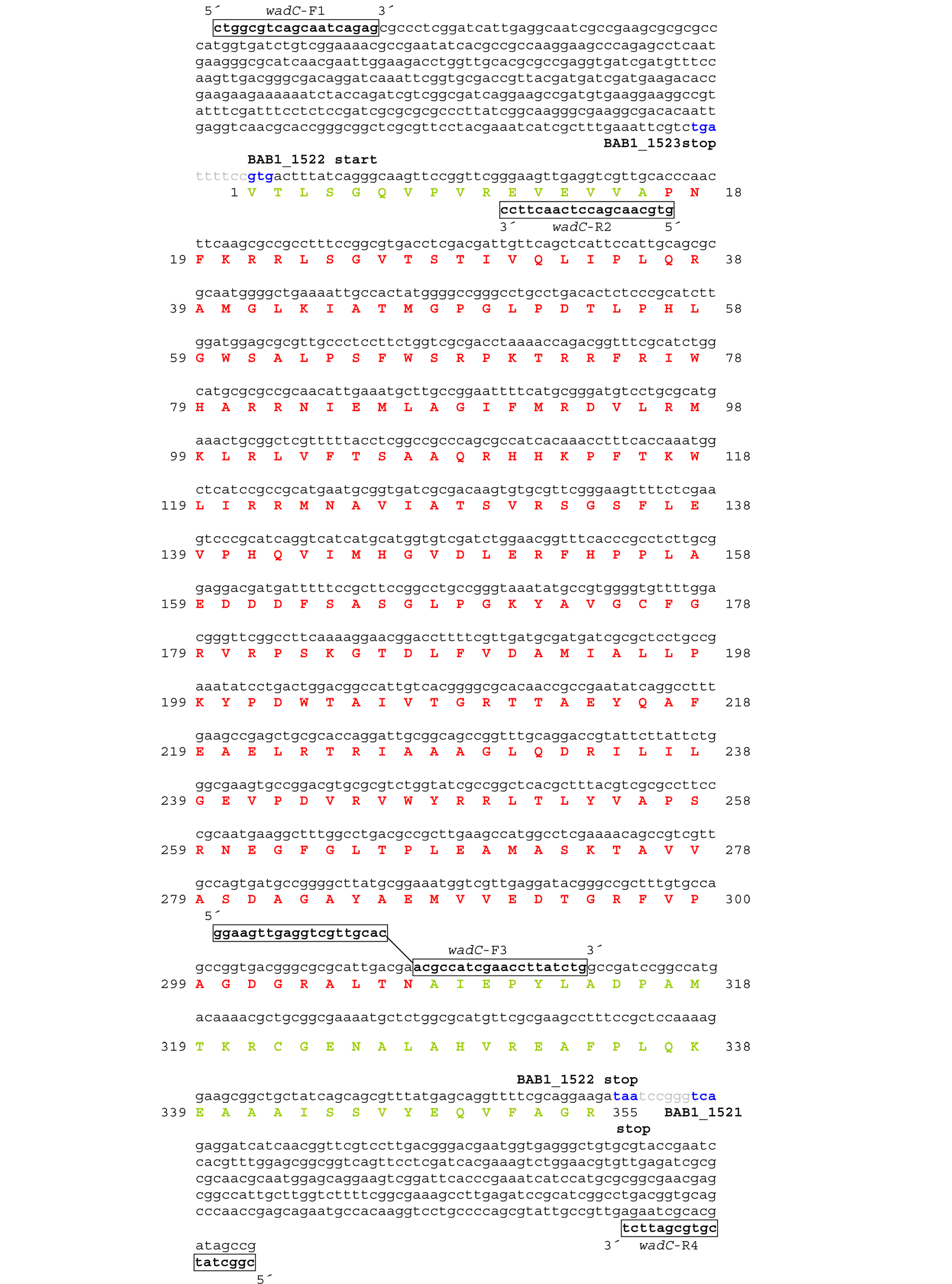

Supplement: Figure S1 — BAB1_1522 (BaΔ wadC ) and its upstream and downstream regions. The DNA sequence is registered at NCBI with accession number YP_414888.1 (Gene ID: 3788779). Start and stop codons are in blue characters; grey characters denote intergenic nucleotides; primers used for mutagenesis are in bold characters; red and green characters mark amino acids deleted and present in BaΔwadC, respectively. (TIF) [file ppat.1002675.s001.tif]

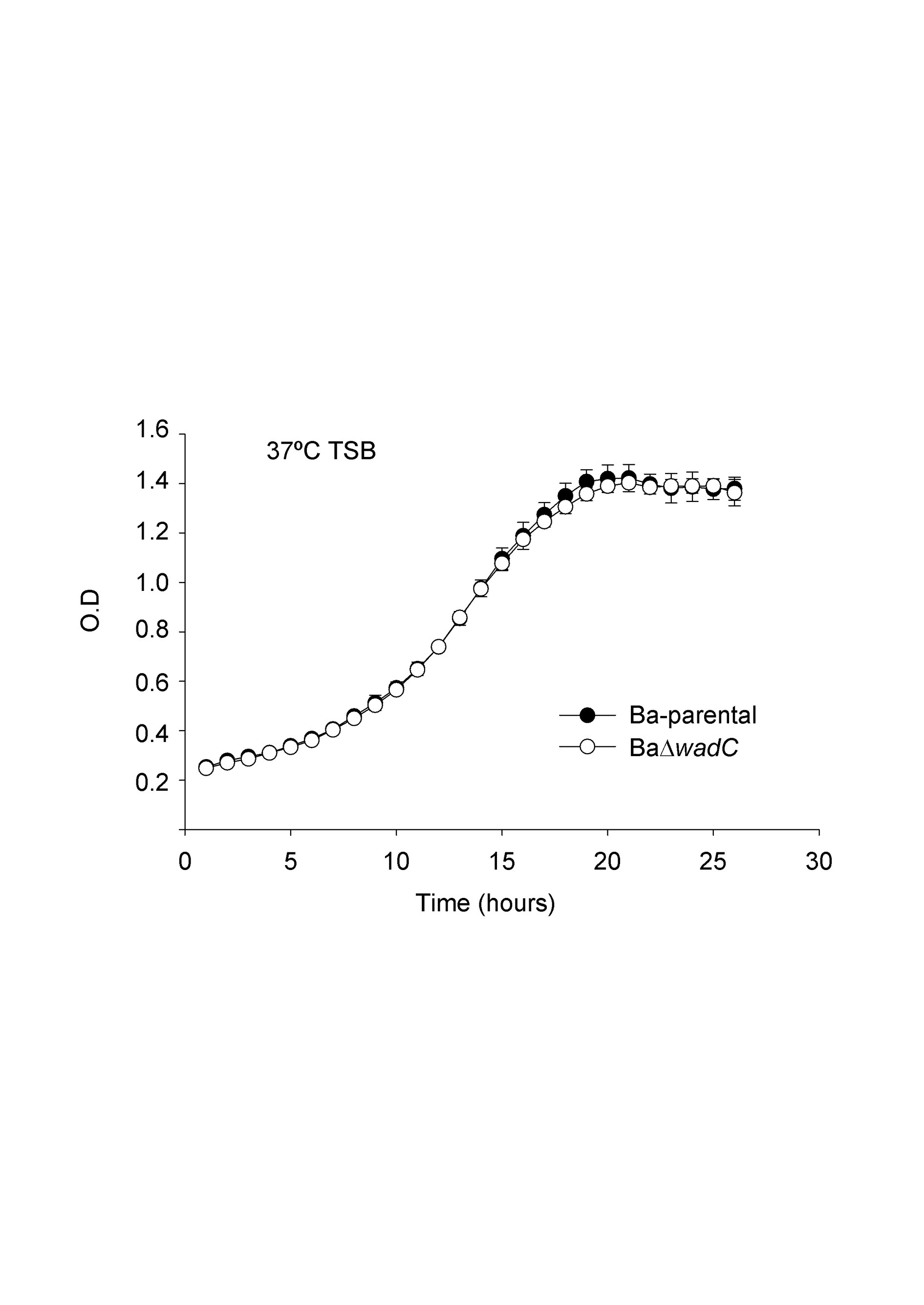

Supplement: Figure S2 — Representative growth curves of Ba-parental and BaΔ wadC in TSB at 37°C. (each point represents the mean of triplicate samples; the experiment was repeated three times with similar results). (TIF) [file ppat.1002675.s002.tif]

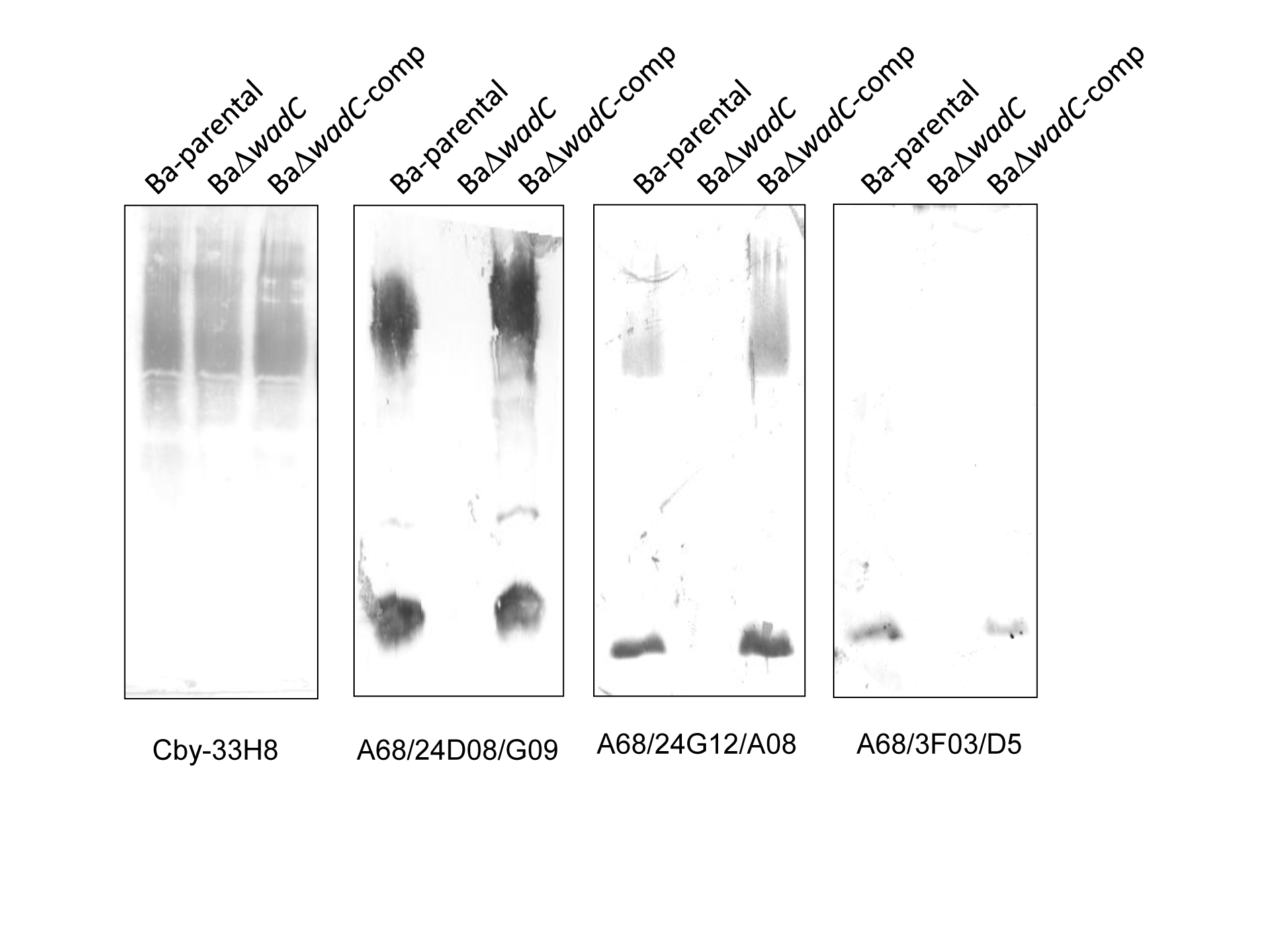

Supplement: Figure S3 — Ba ΔwadC carries a partially defective LPS core oligosaccharide. Western blot analyses were performed with monoclonal antibodies Cby-33H8 (O-polysaccharide C/Y epitope) A68/24D08/G09, A68/24G12/A08 and A68/3F03/D5 (core oligosaccharide) and SDS-proteinase K LPS extracts of Ba-parental, BaΔwadC and BaΔwadC-compl (complemented mutant). (TIF) [file ppat.1002675.s003.tif]

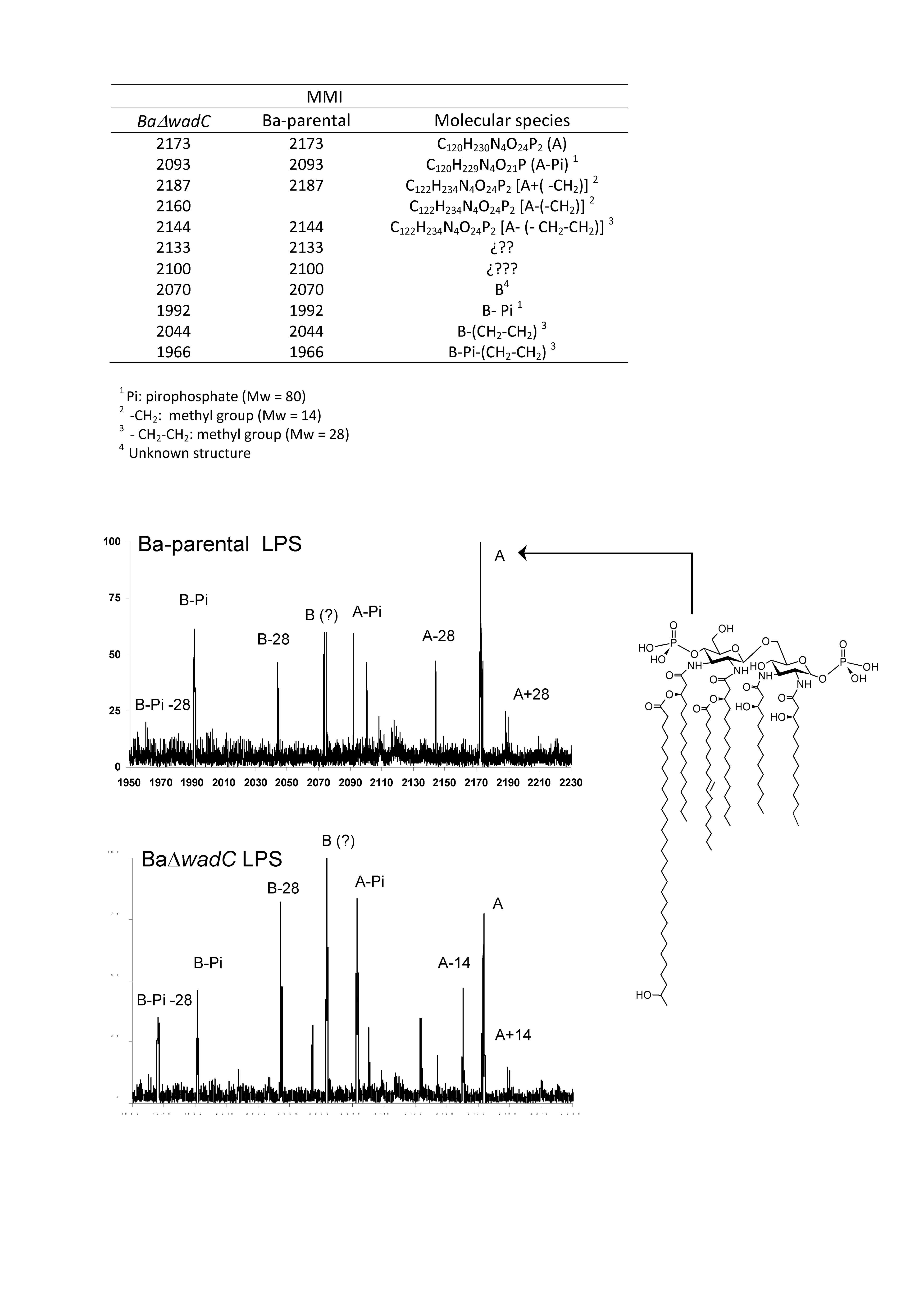

Supplement: Figure S4 — MALDI-TOF analysis of lipid A. The table summarizes the results obtained with several independent preparations, and the figures below show representative spectra (peak B is an uncharacterized molecular species). (TIF) [file ppat.1002675.s004.tif]

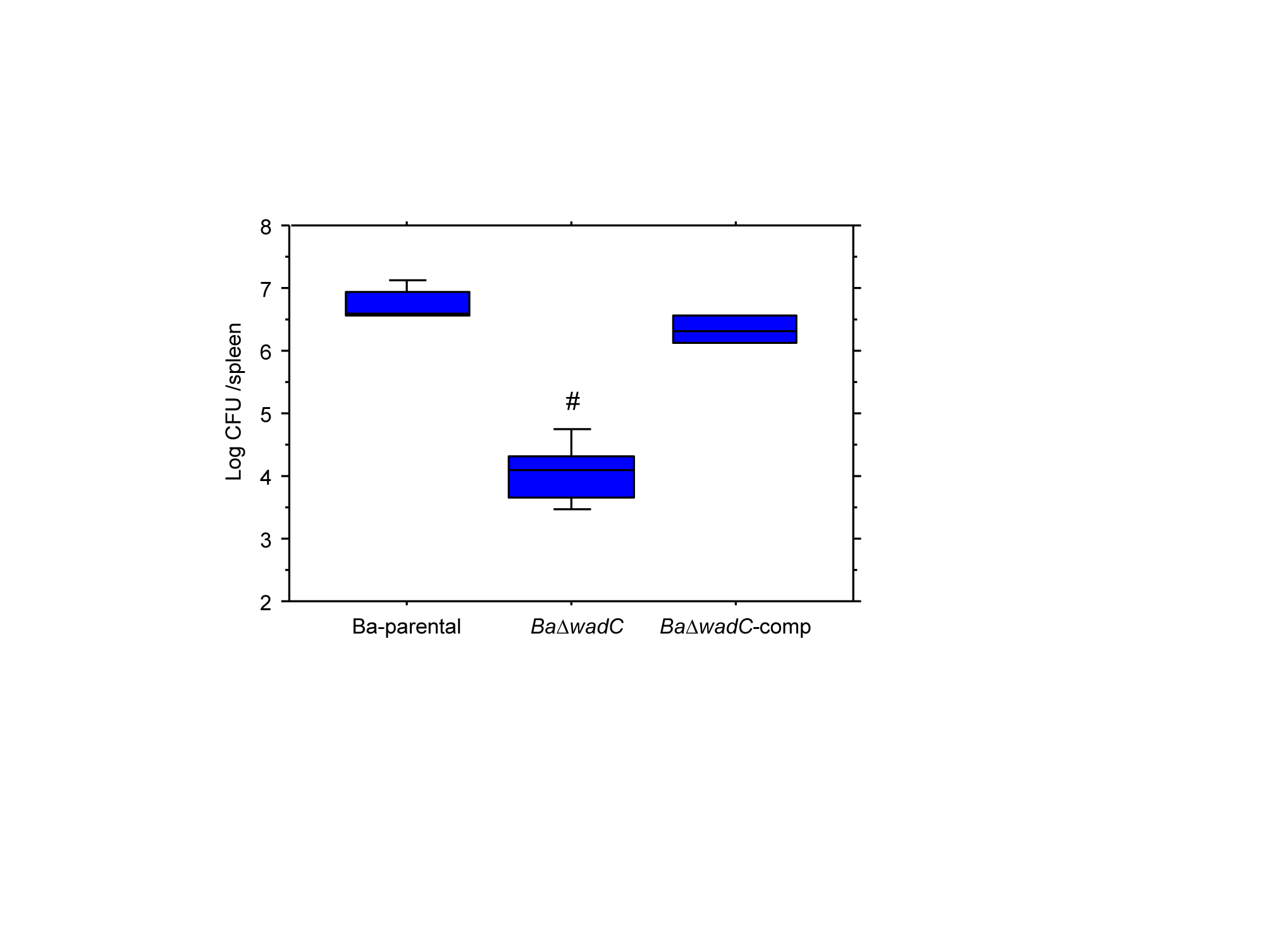

Supplement: Figure S5 — Complementation with p wadC restores the ability of BaΔ wadC to multiply in mice. Mice were inoculated intraperitoneally with 5×10^4^ Ba-parental, BaΔwadC and BaΔwadC-compl (complemented mutant). Number of CFU in spleens was determined eight weeks after inoculation. Differences between BaΔwadC and Ba-parental or BaΔwadC-compl were statistically significant. (#, p<0.001). (TIF) [file ppat.1002675.s005.tif]

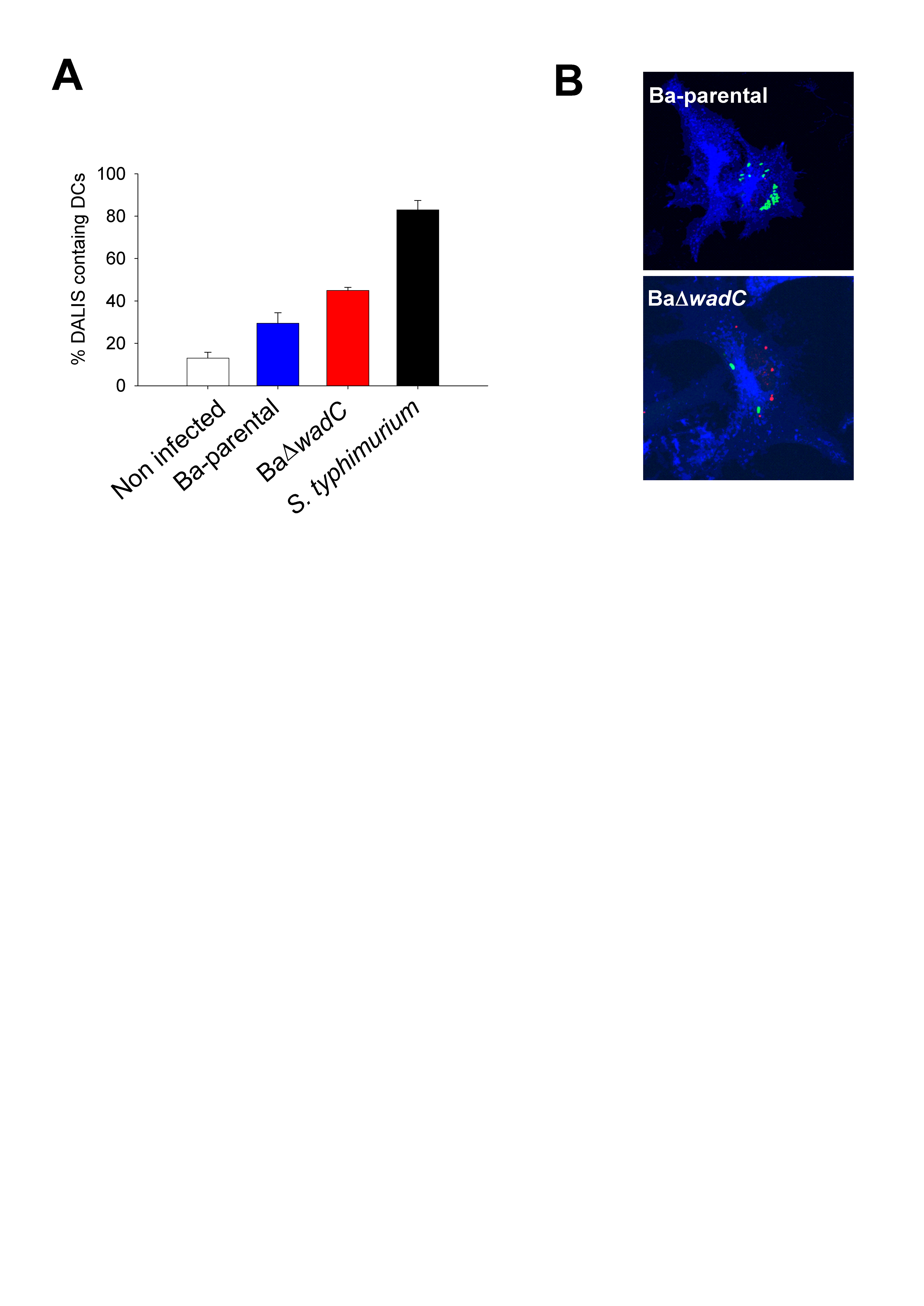

Supplement: Figure S6 — BaΔ wadC induces DALIS in BMDC. (A), Percentages of BMDCs infected with either Ba-parental, BaΔwadC or S. Typhimurium that contain DALIS. (B), representative confocal images of BMDCs infected with Ba-parental GFP or BaΔwadC GFP (in green) labeled with Moabs to CD11c (in blue) and FK2 (in red) 24 hours after infection. (TIF) [file ppat.1002675.s006.tif]

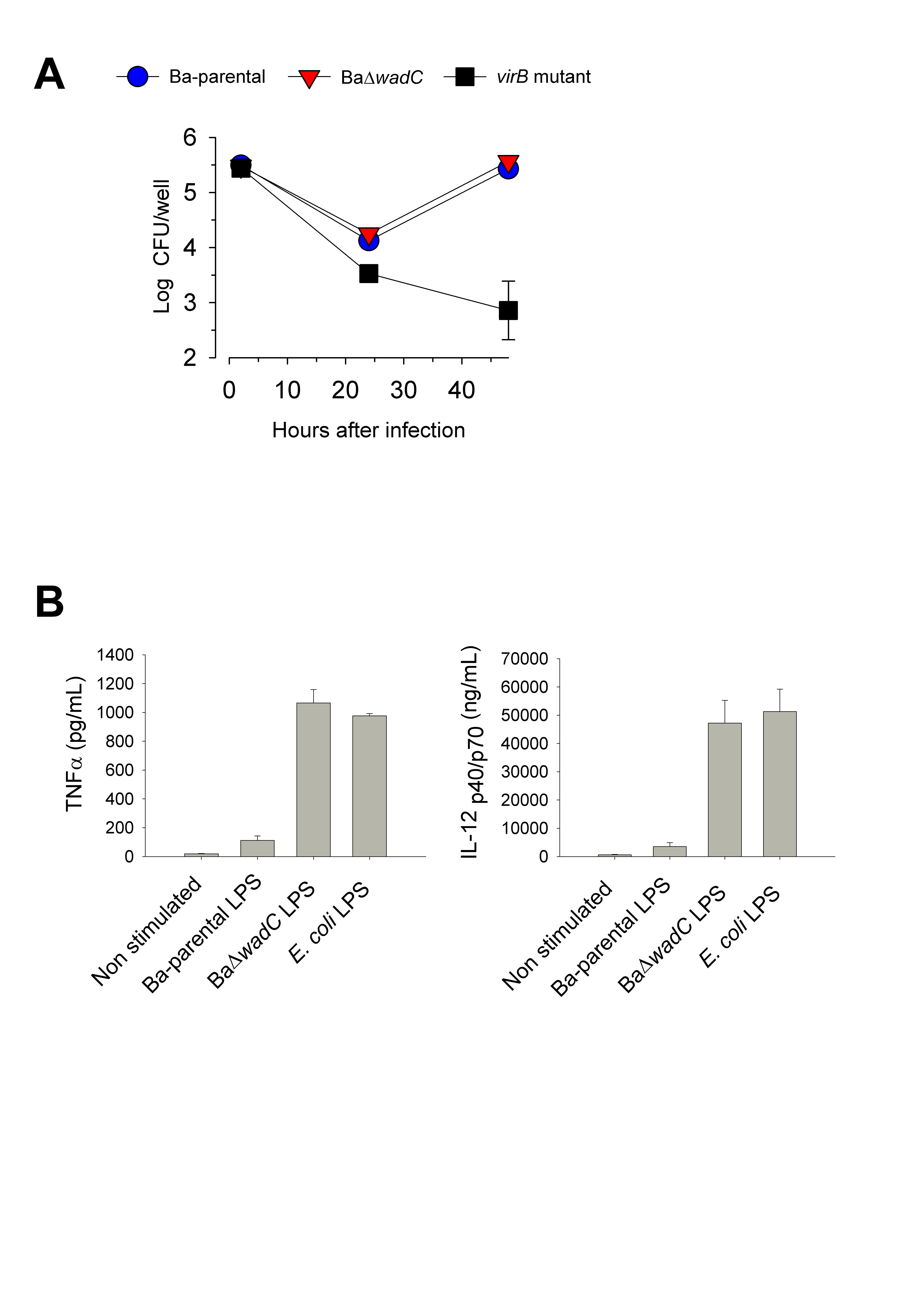

Supplement: Figure S7 — BaΔ wadC LPS induces cytokine release in BMDM. (A), Intracellular replication of Ba-parental, BaΔwadC, BaΔwadC-compl and virB mutant in BMDM. (B), TNF-α (left panel) and IL-12 p40/p70 (right panel) released by BMDM 24 h after incubation with 10 µg/mL of Ba-parental or BaΔwadC LPS, or with 100 ng/mL of E. coli LPS as measured by ELISA. (TIF) [file ppat.1002675.s007.tif]

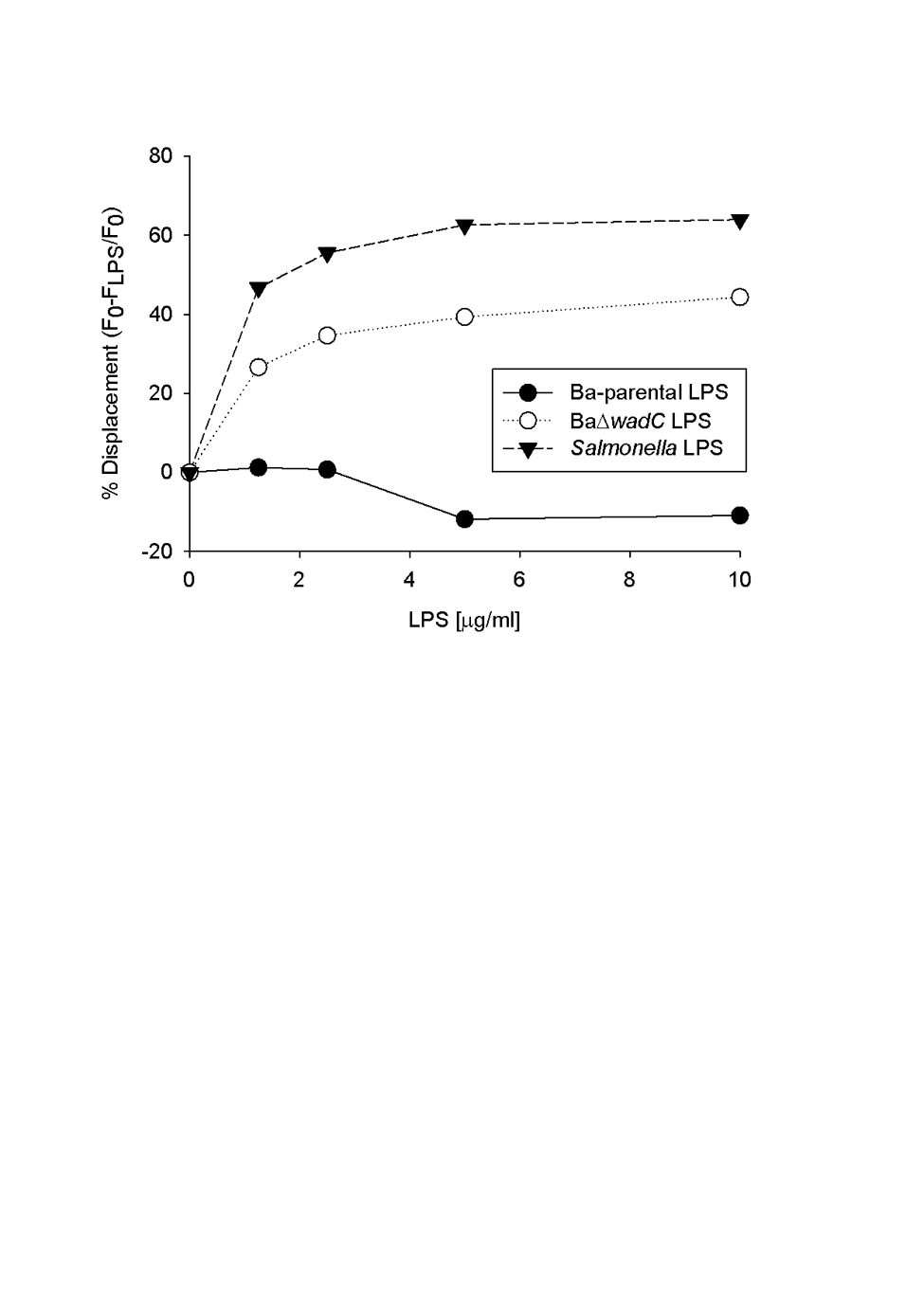

Supplement: Figure S8 — LPS binding to MD-2 assessed by displacement of bis-ANS. The bis-ANS/hMD2 complex (200 nM/200 nM) was incubated for 30 min to reach stable fluorescence (F0). Then, increasing amounts of the indicated LPSs were added and fluorescence (FLPS) measured. Salmonella LPS was used as control. The results shown are representative of three independent experiments. (TIF) [file ppat.1002675.s008.tif]

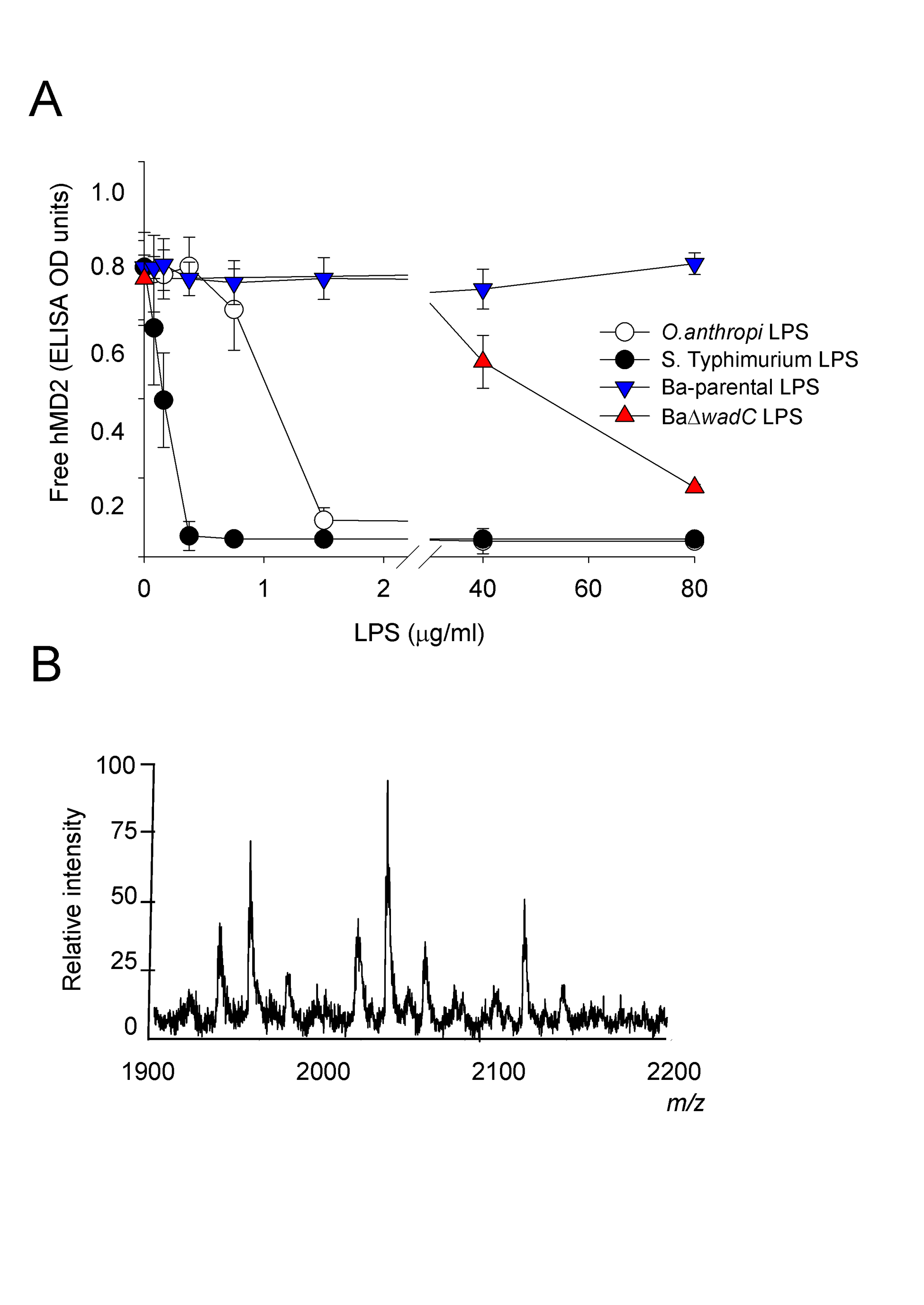

Supplement: Figure S9 — Ochrobactrum anthropi LPS binding to MD-2. (A), After incubation of 0.75 µM hMD-2 with increasing LPS concentrations, the fraction of hMD-2 not bound to LPS was detected with free-hMD-2 specific antibody 9B4 by ELISA. (B), MALDI-TOF analysis of Ochrobactrum anthropi lipid A. (TIF) [file ppat.1002675.s009.tif]

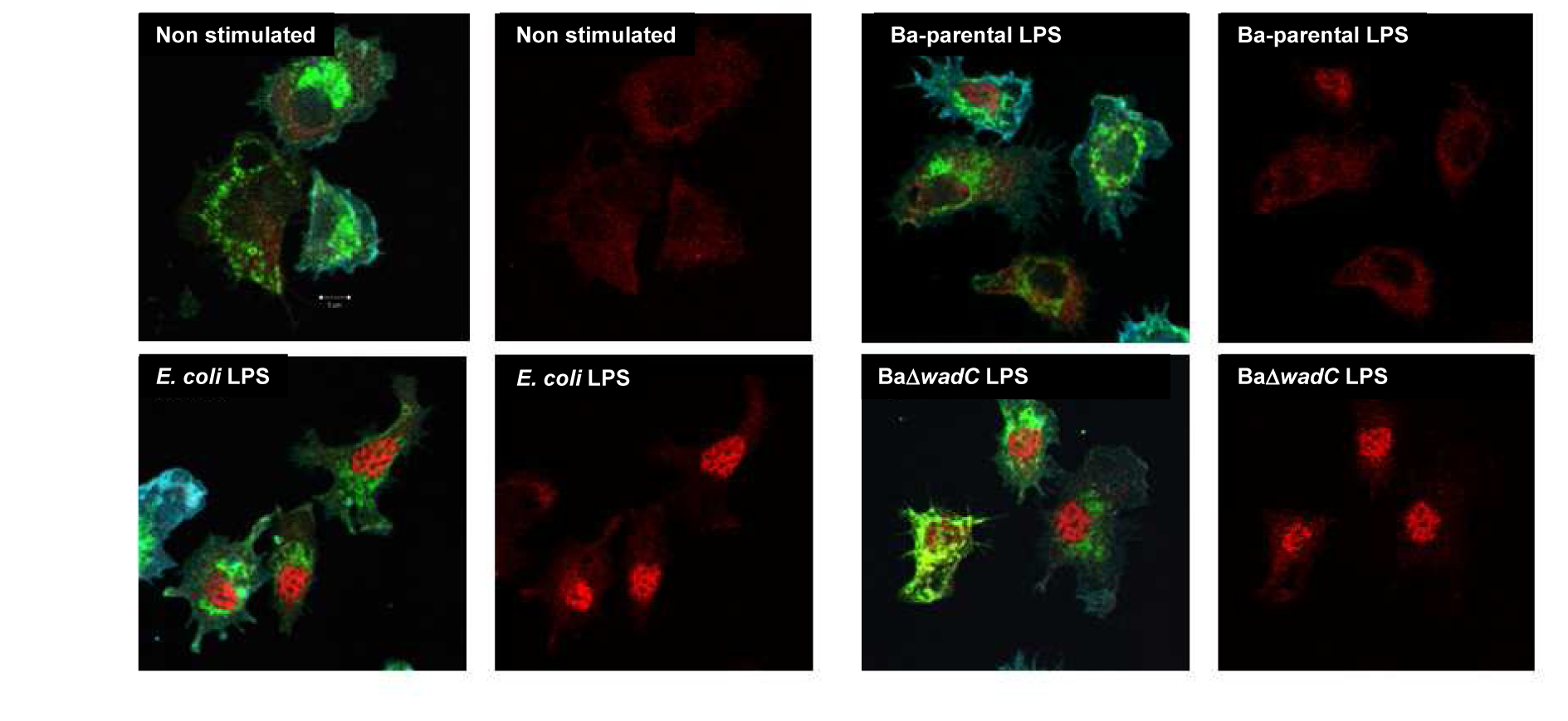

Supplement: Figure S10 — Nuclear translocation of NF-κB subunit in LPS-stimulated BMDC. Cells were stimulated for 1 h with cell culture medium, E. coli LPS (100 ng/mL), Ba-parental LPS (10 µg/mL) or BaΔwadC LPS (10 µg/mL). Cells were stained with CD11c (in blue) and MHCII (in green). The nuclear translocation NF-κB subunit p65/ReiA (in red) was analyzed by confocal microscopy. (TIF) [file ppat.1002675.s010.tif]

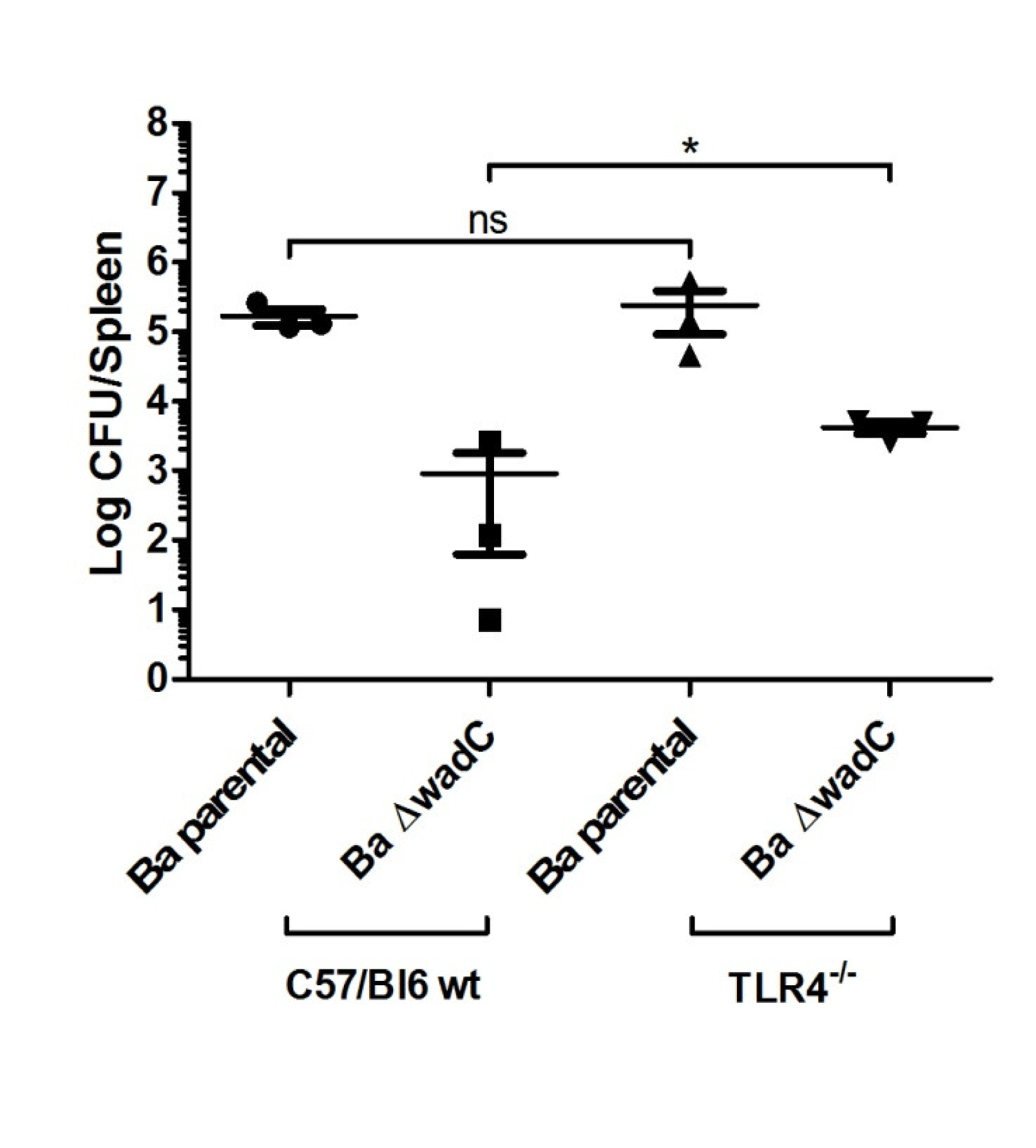

Supplement: Figure S11 — Multiplication of Ba-parental or BaΔ wadC in the spleens of TLR4 KO mice. Mice were intraperitoneally inoculated with 1×10^6^ CFU of either Ba-parental or BaΔwadC. Ten weeks after infection the number of CFU per spleen were determined. (TIF) [file ppat.1002675.s011.tif]
